# SRARP and HSPB7 are epigenetically regulated gene pairs that function as tumor suppressors and predict clinical outcome in malignancies

**DOI:** 10.1002/1878-0261.12195

**Published:** 2018-04-16

**Authors:** Ali Naderi

**Affiliations:** ^1^ Cancer Biology Program University of Hawaii Cancer Center Honolulu HI USA

**Keywords:** 1p36, C1orf64, cancer survival, HSPB7, SRARP, tumor suppressor

## Abstract

Deletions of chromosome 1p36 are common in cancers; however, despite extensive studies, there has been limited success for discovering candidate tumor suppressors in this region. SRARP has recently been identified as a novel corepressor of the androgen receptor (AR) and is located on chromosome 1p36. Here, bioinformatics analysis of large tumor datasets was performed to study *SRARP* and its gene pair, *HSPB7*. In addition, using cancer cell lines, mechanisms of SRARP and HSPB7 regulation and their molecular functions were investigated. This study demonstrated that *SRARP* and *HSPB7* are a gene pair located 5.2 kb apart on 1p36.13 and are inactivated by deletions and epigenetic silencing in malignancies. Importantly, SRARP and HSPB7 have tumor suppressor functions in clonogenicity and cell viability associated with the downregulation of Akt and ERK. *SRARP* expression is inversely correlated with genes that promote cell proliferation and signal transduction, which supports its functions as a tumor suppressor. In addition, AR exerts dual regulatory effects on *SRARP*, and although an increased AR activity suppresses *SRARP* transcription, a minimum level of AR activity is required to maintain baseline *SRARP* expression in AR+ cancer cells. Furthermore, as observed with SRARP, HSPB7 interacts with the 14‐3‐3 protein, presenting a shared molecular feature between SRARP and HSPB7. Of note, genome‐ and epigenome‐wide associations of *SRARP* and *HSPB7* with survival strongly support their tumor suppressor functions. In particular, DNA hypermethylation, lower expression, somatic mutations, and lower copy numbers of *SRARP* are associated with worse cancer outcome. Moreover, DNA hypermethylation and lower expression of *SRARP* in normal adjacent tissues predict poor survival, suggesting that *SRARP* inactivation is an early event in carcinogenesis. In summary, SRARP and HSPB7 are tumor suppressors that are commonly inactivated in malignancies. *SRARP* inactivation is an early event in carcinogenesis that is strongly associated with worse survival, presenting potential translational applications.

**Abbreviations**
AR: androgen receptor
CC: correlation coefficient
CNV: copy number variation
CT: cycle threshold
ER: estrogen receptor
FBS: fetal bovine serum
GBM: glioblastoma multiforme
HDAC: histone deacetylase
ICGC: International Cancer Genome Consortium
IP: immunoprecipitation
MAPK: mitogen‐activated protein kinase
ORF: open reading frame
PCC: Pearson correlation coefficient
PIP: prolactin‐induced protein
qRT‐PCR: quantitative real‐time polymerase chain reaction
RNA‐seq: RNA sequencing
RPKM: reads per kilobase per million mapped reads
RSEM: RNA‐Seq by Expectation Maximization
SRARP: steroid receptor associated and regulated protein
TCGA: The Cancer Genome Atlas
TSA: trichostatin A

## 1 Introduction

Identification and characterization of novel cancer genes are paramount for advancing our understanding of the biology of cancer and discovery of novel therapeutic targets and biomarkers in malignancies. Importantly, large‐scale integrated genomic studies have provided a powerful tool for the discovery and analysis of cancer genes (Lawrence *et al*., 2014; Mo *et al*., 2013). In this respect, a genome‐wide approach has been applied to identify a network of the androgen receptor (AR)‐co‐expressed genes in breast cancer and to discover novel AR target genes and coregulators (Naderi, 2015a, 2017). This approach has recently led to the identification of a novel AR coregulator, SRARP (steroid receptor associated and regulated protein), which is the updated nomenclature for C1orf64 (Naderi, 2017).

Notably, *SRARP* is highly co‐expressed with *AR* in breast cancer cell lines, primary breast tumors, and metastatic breast cancer (Naderi, 2017). *SRARP* also has a relatively higher expression in breast tumors that are estrogen receptor‐positive (ER+), lower grade, and lobular histology (Naderi, 2017; Su *et al*., 2012). Moreover, functional studies identified an interplay between AR and SRARP in breast cancer cells (Naderi, 2017). In this interplay, AR activation directly suppresses *SRARP* transcription, and SRARP, in turn, interacts with AR as a corepressor and negatively regulates AR‐mediated induction of prolactin‐induced protein (PIP) and the reporter activity of androgen response elements (Naderi, 2017). In addition, this corepressor effect of SRARP results in a reduction in AR binding to the *PIP* promoter (Naderi, 2017).

The other aspect of SRARP‐AR interplay involves a cross talk between AR and ER signaling in ER+ cells. In this process, AR activation abrogates ER‐mediated induction of progesterone receptor (PGR). In contrast, SRARP is necessary for *PGR* expression; therefore, the repression of SRARP by AR has an inhibitory effect on the positive regulatory function of SRARP on ER activity (Naderi, 2017). Other studies have suggested that SRARP is also involved in the transcriptional activities of ER and the activation of ER results in the suppression of *SRARP* expression in ER+ breast cancer cells (Luo and Zhang, 2016; Luo *et al*., 2016). Collectively, these findings indicate that *SRARP* is highly co‐expressed with *AR* in breast cancer and has transcriptional regulatory effects on AR and ER signaling.

Furthermore, a combination of bioinformatics analysis and biochemical studies revealed that SRARP is a phosphothreonine protein and an interacting partner of 14‐3‐3 in breast cancer cells (Naderi, 2017). 14‐3‐3 is a chaperone and scaffolding protein that binds serine/threonine‐phosphorylated residues and regulates key proteins involved in various cellular processes such as intracellular signaling and gene transcription (Mackintosh, 2004; Zilliacus *et al*., 2001). In view of these facts, SRARP interaction with 14‐3‐3 may have a role in the molecular functions of SRARP by modulating the chaperone activity of this key protein. Further studies are needed to elucidate SRARP functions in the pathobiology of malignancies and to identify the translational implications of this novel cancer gene.

In this study, a comprehensive investigation of *SRARP* gene transcription, epigenetic regulation, and copy number variation is conducted across malignancies. In addition, SRARP function is examined in the pathobiology of cancer. This study reveals that *SRARP* and its gene pair, *HSPB7*, are epigenetically regulated tumor suppressors and predict clinical outcome in malignancies.

## 2 Materials and methods

### 2.1 Bioinformatics

#### 2.1.1 Copy number correlation analysis in malignancies

The ONCOMINE 4.5 database was used to identify genes that have highly correlated copy numbers with *SRARP* (*C1orf64*) across malignancies of multiple tissue origins (http://www.oncomine.org) (Rhodes *et al*., 2004). Copy number correlation analysis for *SRARP* was carried out in a total of 12 767 samples across 37 different cancer datasets (Table S1). These included a total of 34 datasets from 14 different cancer types in addition to three multicancer cohorts. Next, using log2 copy number units, *SRARP*‐correlated genes were identified at a significance level of *P* ≤ 0.0001 and the highest ranking correlated genes in each dataset were discovered based on the correlation coefficient (CC) cutoff of more than 0.95. For each tumor type, overlapping *SRARP*‐correlated genes were identified and chromosomal location of each gene was found using HUGO Gene Nomenclature Committee (HGNC) online repository (https://www.genenames.org/).

#### 2.1.2 Gene‐based display

The Vertebrate Genome Annotation (VEGA) database was applied to identify the location of *SRARP* (*C1orf64*) gene on chromosome 1 and the distance between genes with correlating copy numbers (http://vega.sanger.ac.uk) (Harrow *et al*., 2014). In addition, a gene‐based display of *SRARP* and *HSPB7* was obtained using VEGA.

#### 2.1.3 Protein motif analysis

scansite 3 software was employed to identify motifs within HSPB7 protein that are likely to be phosphorylated by specific protein kinases or bind to domains such as SH2, 14‐3‐3, or PDZ (http://scansite3.mit.edu/) (Obenauer *et al*., 2003; Yaffe *et al*., 2001). HSPB7 protein sequence was obtained from Ensembl genome browser (http://www.ensembl.org/index.html). Motif scan was carried out with high stringency (best 0.2% of all sites) using HSPB7 sequence. Scansite analysis was performed to identify site of each motif and predicted domain, sequence score, percentile of score compared to all records used in this search, sequence of each motif, and surface accessibility for the predicted sites. HSPB7 and SRARP sequence alignment was examined using NCBI Protein BLAST (https://blast.ncbi.nlm.nih.gov/Blast.cgi?PAGE=Proteins).

#### 2.1.4 Gene expression and promoter methylation profiles in tumors

Gene expression and promoter methylation data for *SRARP* and *HSPB7* genes were analyzed for eighteen tumor types and their respective normal tissue controls from The Cancer Genome Atlas (TCGA) datasets performed by the Office of Cancer Genomics, National Cancer Institute (https://gdc.cancer.gov/) (Grossman *et al*., 2016). Gene expression data were derived from RNA sequencing (RNA‐seq) RPKM (reads per kilobase per million mapped reads) values in TCGA Data Portal using MethHC 1.0.3 (http://methhc.mbc.nctu.edu.tw/php/index.php). Median expression levels were obtained for tumor and normal samples in each dataset. Next, differential gene expression values were calculated as follows: log2 (RPKM + 1)‐transformed median values of tumor−log2 (RPKM + 1) of normal. Median values were applied to create a heat map for the cohort using Microsoft Excel 2013 (Redmond, WA, USA). To calculate *P* values for differential expression between tumor and normal samples, the Mann–Whitney *U*‐test was applied using ibm spss statistics 23 (Armonk, NY, USA).

Promoter methylation analysis for tumor and normal samples were carried out using MethHC on the data obtained from Illumina Infinium HumanMethylation450 BeadChip in TCGA Data Portal. MethHC uses beta value for measuring methylation level ranging from 0 (least methylated) to 1 (most methylated), and methylation level is given by: beta = Methylated probe intensity (M)/(Unmethylated probe intensity (U) + Methylated probe intensity (M) + 100) (Huang *et al*., 2015). Next, promoter methylation ratios of tumor to normal for *SRARP* and *HSPB7* genes were calculated in each tumor type, and a heat map was created to depict changes in the median ratios across the cohort. Statistical significance analysis was conducted to test the difference between tumor and normal samples in each dataset using a *t*‐test after confirming the normal distribution of data. Furthermore, the associations between the promoter methylation and expression values for each gene were measured across all tumor datasets by Pearson correlation coefficient (PCC) and linear regression curve estimation using ibm spss statistics 23.

#### 2.1.5 Gene‐level copy number measurement in malignancies

Copy number data for *SRARP* and *HSPB7* genes across different malignancies were calculated from TCGA datasets (https://gdc.cancer.gov/) (Grossman *et al*., 2016). Public TCGA databases were accessed using the UCSC Xena browser and bioinformatics tool (https://xenabrowser.net/) (Goldman *et al*., 2015). Copy number profiles were measured using whole‐genome microarray at a TCGA genome characterization center. Next, TCGA FIREHOSE pipeline applied the GISTIC2 method to produce segmented copy number variation (CNV) data, which were then mapped to genes to produce gene‐level estimates (Mermel *et al*., 2011). GISTIC2 further thresholded the estimated values to −2, −1, 0, 1, 2, representing copy number deletions, diploid normal copy, and copy number gains. Genes were mapped onto the human genome coordinates using UCSC Xena HUGO probeMap (https://xenabrowser.net/). A total of 35 TCGA datasets across different malignancies were analyzed using the GISTIC2_thersholded method to measure *SRARP* and *HSPB7* gene‐level copy number changes. In addition, TCGA Pan‐Cancer dataset constituting 12 821 samples was also analyzed. The significance levels for copy number changes were calculated using the Kruskal–Wallis test. In addition, mean copy number changes for *SRARP* and *HSPB7* genes were applied to create a heat map.

#### 2.1.6 Functional annotation analysis

An expression microarray dataset in 50 breast cancer cell lines was extracted from a study published by Kao *et al*. (2009). The extracted expression matrix was analyzed to identify genes that were highly correlated with *SRARP* at a PCC cutoff of │CC│ ≥ 0.6, *P* < 0.001, as described before (Naderi, 2017). In this process, two *SRARP* gene signatures were identified based on positive (≥0.6) and inverse (≤−0.6) correlations with *SRARP* expression across the cohort. Next, functional annotation clustering of each signature was carried out using The Database for Annotation, Visualization and Integrated Discovery (DAVID) Bioinformatics Resources (National Institute of Allergy and Infectious Diseases, Bethesda, MD, USA) (Huang da *et al*., 2009a,b).

#### 2.1.7 *SRARP*‐co‐expressed genes in breast and prostate cancers

Genes that are highly co‐expressed with SRARP in breast and prostate cancers were identified using the ONCOMINE 4.5 database. Co‐expression analysis for *SRARP* was carried out across 28 breast cancer expression microarray datasets with a total of 5128 tumors and 5 prostate cancer datasets with a total of 222 samples. Each dataset was analyzed separately to identify *SRARP*‐co‐expressed genes at a CC cutoff of >0.6, *P* ≤ 0.0001. CC values were derived from the average linkage hierarchical clustering calculated from the correlation value of the node at which the expression of *SRARP* and that of its co‐expressed genes were joined. The node correlation value was computed as the average of all pairwise correlations among genes included at the node. Next, *SRARP*‐co‐expressed gene sets were compiled in each cancer type. Finally, functional annotation clustering of each combined gene set was performed using DAVID.

#### 2.1.8 Survival analysis

The Cancer Genome Atlas Pan‐Cancer datasets were analyzed to examine the association of *SRARP* and *HSPB7* methylation, expression, and mutations with survival. TCGA datasets were accessed using the UCSC Xena browser and bioinformatics tool (https://xenabrowser.net/). Duplicate samples were removed from the datasets before conducting survival analysis for primary tumors. In addition, TCGA data from normal solid tissues were separately analyzed.

For DNA methylation analysis, TCGA Pan‐Cancer DNA methylation 450K array beta values were compiled by combining the data from all TCGA cohorts measured using the Illumina Infinium HumanMethylation450 platform. To analyze exon expression, TCGA Pan‐Cancer exon expression was measured using the Illumina HiSeq technology and data from all TCGA cohorts were combined to produce the dataset. In this analysis, expression values are log2 (RPKM + 1)‐transformed exon‐level transcription estimates in RPKM values. In addition, gene expression data were obtained using TCGA Pan‐Cancer RNA‐seq results in which expression values are log2(*x* + 1)‐transformed RSEM values (RSEM: RNA‐Seq by Expectation Maximization). TCGA Pan‐Cancer somatic mutation data were compiled using all TCGA cohorts, and the calls were generated at Broad Institute Genome Sequencing Center using the MuTect method (Cibulskis *et al*., 2013). MuTect calls from TCGA cohorts were combined to produce the mutation dataset.

Moreover, Pan‐Cancer datasets from International Cancer Genome Consortium (ICGC) were accessed using the UCSC Xena browser and ICGC Data Portal (https://dcc.icgc.org/). ICGC datasets were applied to further assess the association of *SRARP* and *HSPB7* gene expression with survival in patients with cancer using donor centric data. The datasets were also separately analyzed for normal adjacent tissues. Gene expression results were obtained using RNA‐seq in which expression units are log2 (ICGC‐normalized read count + 1e‐8) values. Furthermore, survival analysis was carried out based on *SRARP* and *HSPB7* copy numbers in ICGC cohorts. Copy numbers were assayed by Illumina HiSeq from all available ICGC projects and the results were converted to log2 (tumor/normal) values.

Survival analysis was conducted using Kaplan–Meier curves and the log‐rank test with the application of UCSC Xena bioinformatics tool to estimate the survival probability based on DNA methylation, expression, somatic mutations, and copy numbers of *SRARP* and *HSPB7* genes.

### 2.2 Cell lines and culture

Cell lines were obtained from the European Collection of Authenticated Cell Cultures (ECACC) through Sigma‐Aldrich (St. Louis, MO, USA) and the NCI‐60 collection through the High‐Throughput Facility at the University of Hawaii Cancer Center. Cell lines were authenticated using STR DNA profiles and were tested free from mycoplasma contamination. Cell lines were initially grown and cryopreserved into aliquots, and only low‐passage cells were used for experiments. Culture media were obtained from Life Technologies (Grand Island, NY, USA). Breast cancer cell lines T‐47D (ER+/AR+), MDA‐MB‐231 (ER−/AR−), and MDA‐MB‐468 (ER−/AR−) and endometrial adenocarcinoma cell line Ishikawa were cultured in DMEM/F12 medium supplemented with 10% fetal bovine serum (FBS) (Fisher Scientific, Waltham, MA, USA). Breast cancer cell line MFM‐223 (ER−/AR+) and osteosarcoma cell line U‐2 OS were cultured in DMEM and McCoy′s 5A media, respectively, supplemented with 10% FBS. Renal cell carcinoma cell lines 786‐0 and A498, prostate cancer cell line DU‐145, melanoma cell line UACC‐257, ovarian cancer cell line IGROV1, non‐small‐cell lung carcinoma cell line A549, colorectal adenocarcinoma cell line HCT‐15, and glioblastoma multiforme (GBM) cell line SF‐268 were cultured in RPMI‐1640 medium supplemented with 10% FBS. All cell cultures were performed in a 37 °C incubator with 5% CO2. AR inhibition with enzalutamide was performed at 10 μm concentration for 72 h in full media (Selleck Chemicals, Houston, TX, USA), and an equal volume of solvent only was applied for controls.

### 2.3 Western blot analysis

Rabbit polyclonal SRARP (C1orf64) antibody (Novus Biologicals, Littleton, CO, USA) and rabbit monoclonal HSPB7 antibody (Abcam, Cambridge, MA, USA) were applied at 1 : 250 and 1 : 1000 dilutions, respectively. Rabbit polyclonal 14‐3‐3 (pan) antibody (Millipore, Temecula, CA, USA) was used at a 1 : 5000 dilution. Rabbit monoclonal antibodies for ERK1/2, phospho‐ERK1/2 (Thr202/Try204), Akt (pan), and phospho‐Akt (Thr308) were obtained from Cell Signaling Technology (Danvers, MA, USA) and applied at 1 : 1000 dilutions. Mouse monoclonal α‐tubulin antibody (Sigma‐Aldrich) was applied at a 1 : 2000 dilution to assess loading. Protein concentrations were measured using the BCA Protein Assay Kit (Thermo Fisher Scientific, Waltham, MA, USA), and a total of 30 μg of each cell lysate was used for western blotting. Western blot imaging and analysis of band densities were performed by a C‐DiGit Blot Scanner (LI‐COR, Lincoln, NE, USA). Experiments were performed in three replicates, and mean fold changes are presented.

### 2.4 RNA extraction and quantitative real‐time polymerase chain reaction

RNA extraction was carried out using RNeasy Mini Kit (Qiagen, Valencia, CA, USA). *SRARP* and *HSPB7* gene expression levels were assessed by quantitative real‐time polymerase chain reaction (qRT‐PCR). TaqMan Gene Expression Assays (Life Technologies) for *SRARP* (assay ID: Hs00698851_m1), *HSPB7* (assay ID: Hs00205296_m1), and *AR* (Hs00171172_m1) were applied for qRT‐PCR as instructed by the manufacturer. Housekeeping gene *RPLP0* (Life Technologies) was used as control. Fold change in gene expression is gene expression in the treated group/average gene expression in the control group (Naderi, 2015a; Naderi and Meyer, 2012; Naderi and Vanneste, 2014).

### 2.5 Heat shock induction and hypoxia in cell culture

Heat shock induction in cell lines were carried out as described before (Graner *et al*., 2007). Cells were first overlaid with prewarmed 42 °C media and then incubated at 42 °C for 1 h. After heat shock, the media were replaced with 37 °C media and cells were allowed to recover for 2 h at a 37 °C incubator. For control experiments, cells were overlaid with prewarmed 37 °C media. Induction of hypoxia by CoCl2 solution was performed as previously published (Wu and Yotnda, 2011). Cobalt (II) chloride hexahydrate, which is a chemical inducer of hypoxia‐inducible factor‐1, was obtained from Sigma‐Aldrich. CoCl2 was applied at 100 μm concentration in media, and cells were cultured for 24 h at 37 °C to induce hypoxia. Control experiments were conducted by the addition of solvent alone to media.

### 2.6 Inhibition of DNA methylation and histone deacetylation in cell lines

Demethylation was induced in cancer cell lines with 5‐aza‐2′‐deoxycytidine (5‐aza‐dC) (Millipore) as described before (Mossman *et al*., 2010; Zhang *et al*., 2007). Cells were incubated with 5‐aza‐dC at 10 μm concentration for 72 h, and the culture media were replaced every 24 h with fresh media containing 5‐aza‐dC. Control experiments were performed by the addition of DMSO solvent (Sigma‐Aldrich) and following the same procedure. Cell line treatments with histone deacetylase (HDAC) inhibitor trichostatin A (TSA) (Selleck Chemicals, Houston, TX, USA) were carried out at 1 μm concentration for 24 h as previously published (Gill *et al*., 2013). Control experiments were treated by the addition of solvent alone. Following the completion of 5‐aza‐dC and TSA treatments, RNA from each sample was extracted for qRT‐PCR assays. Experiments were performed in four replicates.

### 2.7 RNA interference

Androgen receptor silencing by RNA interference in T‐47D and MFM‐223 cell lines was carried out by the reverse transfection method using Lipofectamine RNAiMAX (Life Technologies) as previously published (Naderi, 2017). AR silencing was performed using an *AR*‐siRNA duplex (Sigma‐Aldrich): sense, CCAUCUUUCUGAAUGUCCU[dT][dT]; antisense, AGGACAUUCAGAAAGAUGG[dT][dT]. Transfections of siRNA Universal Negative Control # 1 (Sigma‐Aldrich) were used as control. The effect of siRNA silencing was assessed 72 h after transfections.

### 2.8 Transfection of cDNA vectors and generation of stable cell lines

Steroid receptor associated and regulated protein and HSPB7 open reading frame (ORF) clones in pReciever‐M02 plasmids were obtained from GeneCopoeia (Rockville, MD, USA). An empty pReciever‐M02 plasmid was applied for the control experiments. Transfection of each construct was carried out using TurboFect Transfection Reagent (Thermo Fisher Scientific) as instructed by the manufacturer. The overexpression of SRARP and HSPB7 proteins was confirmed by western blotting 48 h after transfection of each expression construct. To generate stable cell lines, 48 h following each transfection, cells were cultured in medium containing G418 (Life Technologies) at 500 μg·mL^−1^ for 21 days.

### 2.9 Clonogenic assay

To investigate clonogenicity, 48 h after transfections, a total of 1000 cells transfected with each cDNA clone containing *SRARP* (SRARP+), *HSPB7* (HSPB7+), or an empty plasmid (CTL‐VEC) were seeded in 6‐well plates and cultured for 21 days in medium containing G418 at 500 μg·mL^−1^ concentration. Every 3 days, the medium was replaced with fresh medium containing the selection drug. After 21 days, colonies were fixed with ice‐cold 100% methanol and stained with 0.5% crystal violet solution in 25% methanol. Colonies containing more than 50 cells were counted using an inverted microscope. Experiments were performed in four replicates.

### 2.10 Cell viability assay

To examine cell viability, MTT assay was carried out on stably transfected SRARP+ and HSPB7+ cell lines using Vybrant MTT Proliferation Assay Kit (Life Technologies). Stable transfections of an empty plasmid were used as controls. Stable lines were seeded at 5000 cells per well in a 96‐well plate and cultured for 72 h followed by MTT assay as instructed by the manufacturer. Experiments were performed in eight replicates.

### 2.11 Co‐immunoprecipitation

Immunoprecipitation (IP) assay for endogenous 14‐3‐3 protein was carried out as previously published (Naderi, 2017). MDA‐MB‐231 cells were transfected with a HSPB7 ORF clone (GeneCopoeia) in 6‐cm dishes using TurboFect Transfection Reagent (Thermo Fisher Scientific). Forty‐eight hours following transfections, each dish was lysed in 0.5 mL of IP lysis buffer supplemented with protease and phosphatase inhibitors (Sigma‐Aldrich). Lysates from two 6‐cm dishes were combined and applied for each set of the 14‐3‐3 IP and control IP experiments. Next, 14‐3‐3 IP was performed using 5 μg of a rabbit polyclonal 14‐3‐3 (pan) antibody (Millipore). Control experiment was conducted with a nonspecific rabbit IgG. Following the 14‐3‐3 IP, supernatants were collected and applied for western blot analysis using HSPB7 and 14‐3‐3 antibodies. In addition, for each sample, 5% of lysate was collected before IP to assess input by western blot analysis using a 14‐3‐3 (pan) antibody. Co‐IP experiments were performed in three replicates.

### 2.12 Statistical analysis

Biostatistics was carried out using ibm spss statistics 23. Student's *t*‐test, paired‐samples *t*‐test, and ANOVA with Dunnett's *post hoc* test for multiple comparisons were applied to calculate the statistical significance between biological replicate experiments. Regression analysis by logarithmic and inverse models was used for the prediction of *SRARP* and *HSPB7* gene expression levels based on the data from the reversal of DNA methylation and histone deacetylation in cancer cell lines. All error bars depict ± SEM.

## 3 Results

### 3.1 *SRARP* and *HSPB7* are gene pairs with closely correlated copy numbers

**Figure 1:**
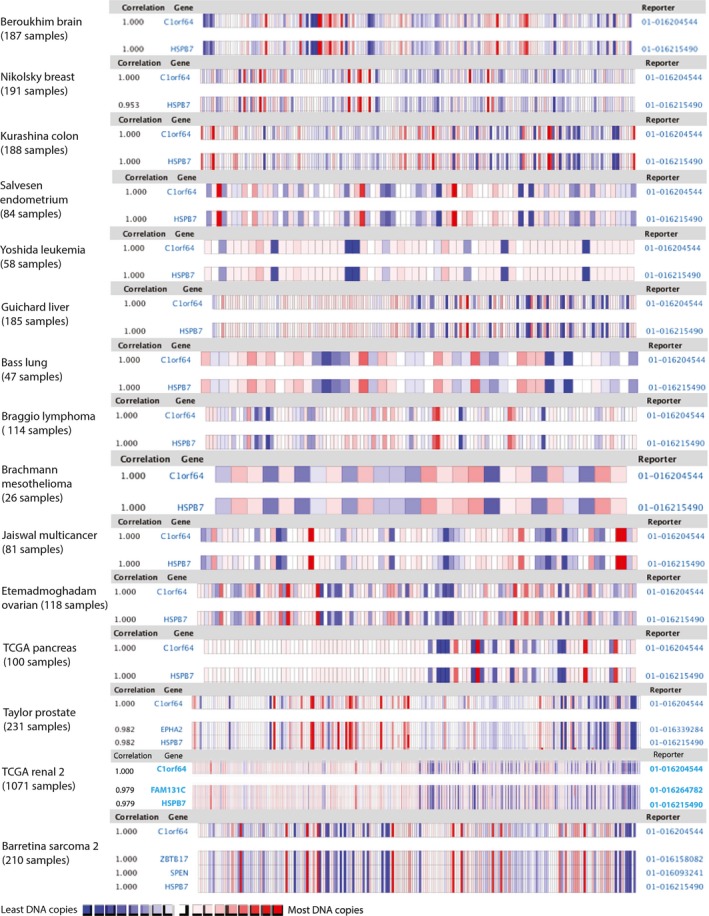
Copy number correlations of *SRARP* and *HSPB7* genes in malignancies. Heat maps and correlation coefficients for *SRARP* (*C1orf64*) and *HSPB7* copy numbers are shown across malignancies of multiple tissue origins. Copy number data were analyzed using the ONCOMINE database. Each heat map depicts the correlation pattern between *SRARP* and *HSPB7* across one of the datasets. Study name, tissue of origin, and sample size are shown for each dataset. Red and blue colors denote higher and lower DNA copies, respectively.

To gain insight into the genomic network of *SRARP*, the list of genes that highly correlate with the copy number of *SRARP* were identified across malignancies of multiple tissue origins. Copy number correlation analysis for *SRARP* was carried out using the ONCOMINE database in a total of 12 767 samples across 37 cancer datasets as explained in methods (Table S1). The highest ranking correlated genes in each dataset were discovered based on the CC cutoff of >0.95 (Table S2). Next, the top ten ranking genes across datasets, which had the highest frequencies of copy number correlations with *SRARP* at a CC of >0.95, were identified (Table 1). Notably, *HSPB7* showed the strongest copy number correlation pattern with *SRARP* across the datasets (Table 1 and Fig. 1). In this respect, *HSPB7* and *SRARP* had a CC value of >0.95 in all 37 analyzed datasets and a CC value of 1 in the majority of malignancies (Table S1 and Fig. 1). The remaining nine genes demonstrated CC values of > 0.95 with *SRARP* in 30 to 34 datasets and included *FAM131C*,* ZBTB17*,* EPHA2*,* CLCNKA*,* CLCNKB*,* SPEN*,* FBLIM1*,* TMEM82*, and *SLC25A34* (Tables 1 and S2).

**Table 1:** List of the top ten ranking genes that have the highest frequencies of copy number correlations with *SRARP* in malignancies at a correlation coefficient (CC) cutoff of >0.95. Copy number correlation analysis for *SRARP* was carried out in a total of 12 767 samples in 37 different cancer datasets using the ONCOMINE 4.5 database. The number of datasets for each *SRARP*‐correlated gene at a CC cutoff of >0.95, chromosomal location of each gene, and the distance between *SRARP* and each gene in kilobases (kb) are presented

Gene name	Number of datasets (CC > 0.95)	Chromosomal location	Distance to *SRARP*
*HSPB7*	37	1p36.13	5.2 kb
*FAM131C*	34	1p36.13	49 kb
*ZBTB17*	34	1p36.13	28 kb
*EPHA2*	33	1p36.13	116 kb
*CLCNKA*	32	1p36.13	10 kb
*CLCNKB*	30	1p36.13	35 kb
*SPEN*	30	1p36.21‐p36.13	64 kb
*FBLIM1*	30	1p36.21	218 kb
*TMEM82*	30	1p36.21	256 kb
*SLC25A34*	30	1p36.21	263 kb

**Figure 2:**
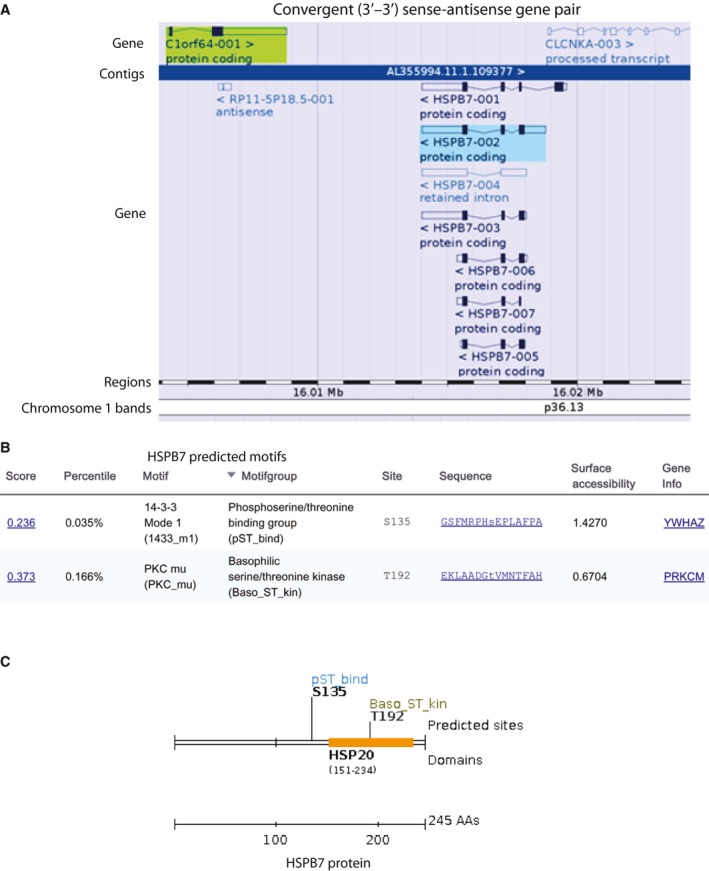
Chromosomal location of *SRARP* and *HSPB7* genes and the predicted motifs of HSPB7 protein. (A) Gene‐based display and the distance between *SRARP* (*C1orf64*) and *HSPB7* genes on chromosome 1p36.13 using VEGA database. *SRARP* and *HSPB7* are shown on the sense and antisense strands, respectively, and the known isoforms for *HSPB7* are demonstrated. *CLCNKA*‐003 start site on the sense strand is also depicted. (B and C) HSPB7 protein sequence was analyzed using scansite 3 software to identify regulatory motifs. Motif scan was carried out with high stringency to detect the best 0.2% of all sites. (B) Predicted motifs and their sequence score, percentile, sequence of motif, and surface accessibility are shown. (C) Predicted motif sites and a HSP20 domain within the HSPB7 sequence. AAs: amino acids.

To investigate the underlying mechanism of *SRARP* gene‐level correlation pattern, chromosomal locations of the top ten ranked genes and their distance to the *SRARP* gene locus were examined. Of note, all the top ten *SRARP*‐correlated genes were located on chromosome 1p36.13 or 1p36.21 with distances between 5.2 and 263 kb to the *SRARP* locus on chromosome 1p36.13 (Tables 1 and S2). Importantly, *HSPB7* shows the closest distance to *SRARP* at only 5.2 kb and these two genes demonstrate a convergent (3′‐3′) pattern of gene pairs with *SRARP* and *HSPB7* located on the sense and antisense strands on chromosome 1p36.13, respectively (Table 1 and Fig. 2A). In addition, all the other *SRARP*‐correlated genes that have a CC value of >0.95 across datasets are also located on chromosome 1p36.13 or 1p36.21 (Table S2). These findings suggest the chromosomal proximity as the main factor in determining a close copy number pattern with *SRARP* in malignancies.

It is known that gene pairs share gene ontology terms suggesting functional correlation (Arnone *et al*., 2012; Krom and Ramakrishna, 2008). Therefore, possible similarities between SRARP and HSPB7 in protein sequence and interacting motifs were investigated. Alignment of SRARP and HSPB7 sequences did not show a significant similarity (E value: 2). Next, HSPB7 sequence was examined using scansite 3 software to identify motifs that are likely to be phosphorylated by specific protein kinases or bind to domains such as SH2, 14‐3‐3, or PDZ. This search, which was carried out with high stringency to detect the best 0.2% of all sites, identified two motifs within the HSPB7 sequence. The first motif was 14‐3‐3 Mode 1, a phosphoserine/threonine binding group (pST_bind), which was predicted to interact with HSPB7 at S135 site with a motif score of 0.236 in the top 0.035% of all sites (Fig. 2B). Notably, it was predicted that GSFMRPH**S**EPLAFPA sequence within the HSPB7 protein may interact with 14‐3‐3. The other predicted HSPB7 motif was PKC mu, a basophilic serine/threonine kinase, which may interact with HSPB7 at T192 site with a score of 0.373 in the top 0.166% of all sites (Fig. 2B). Furthermore, it was predicted that HSPB7 protein contains a HSP20 domain at its 151‐ to 234‐amino acid region (Fig. 2C).

Therefore, we can conclude that *SRARP* and *HSPB7* are gene pairs with closely correlated copy numbers across malignancies of multiple tissue origins. In addition, similar to SRARP, there is a predicted 14‐3‐3 motif within the HSPB7 protein sequence.

### 3.2 *SRARP* and *HSPB7* expression levels are highly regulated by epigenetic silencing

**Figure 3:**
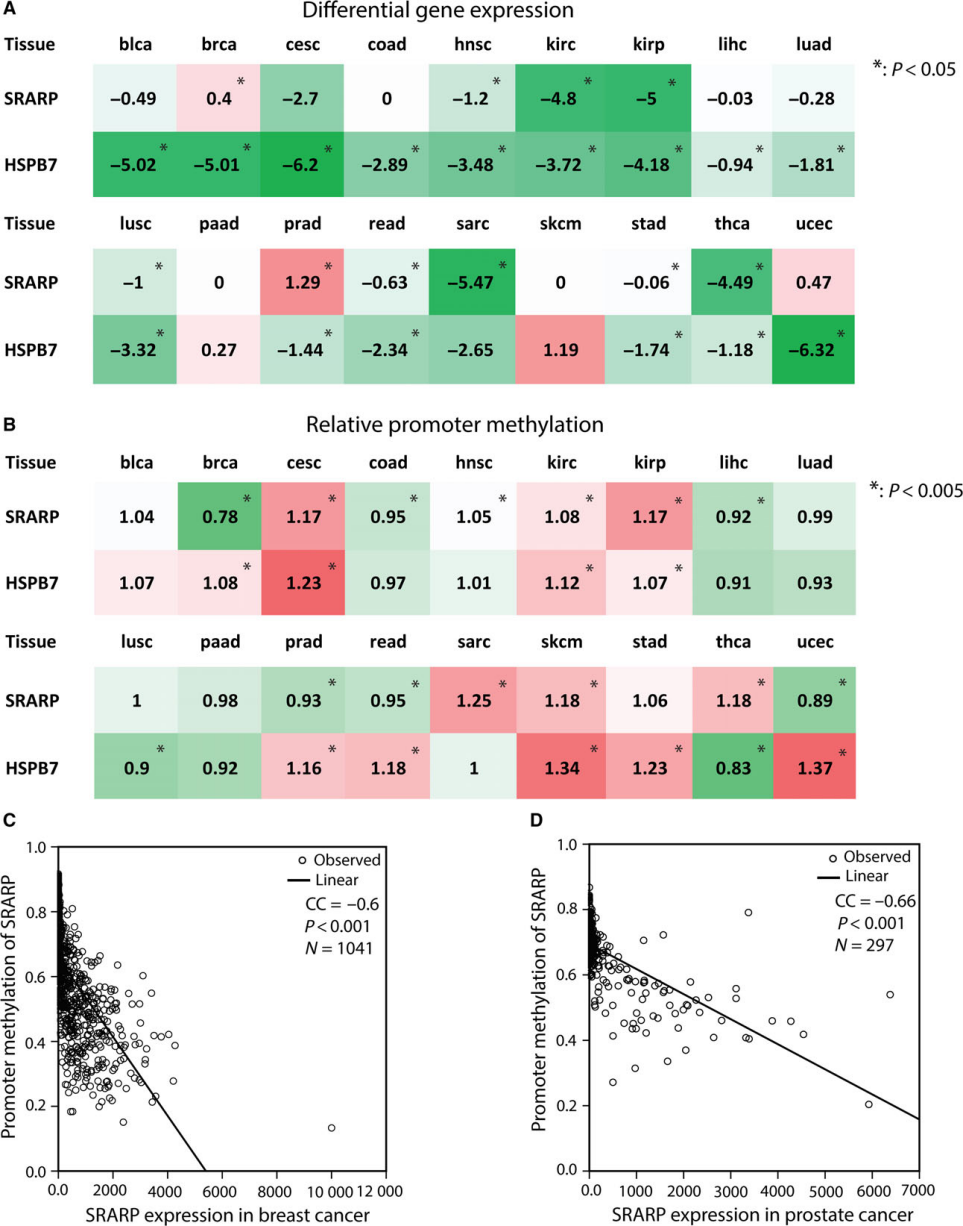
Differential gene expression and relative promoter methylation of *SRARP* and *HSPB7*. (A) Heat map to show *SRARP* and *HSPB7* differential expression values in eighteen tumor types and their matched normal tissues. Differential gene expression values were calculated as follows: log2 (RPKM+1)‐transformed median values of tumor ‐ log2 (RPKM+1) of normal. *P* values for tumor and normal pairs were calculated using the Mann–Whitney *U*‐test. *P < 0.05. Green and red colors denote decrease and increase in differential gene expression, respectively; blca: bladder cancer (CA); brca: breast CA; cesc: cervical CA; coad: colon CA; hnsc: head and neck CA; kirc: renal clear cell CA; kirp: renal papillary CA; lihc: liver CA; luad: lung adeno‐CA; lusc: lung squamous CA; paad: pancreatic CA; prad: prostate CA; read: rectal CA; sarc: sarcoma; skcm: skin melanoma; stad: stomach CA; thca: thyroid CA; ucec: endometrial CA. (B) Heat map to demonstrate *SRARP* and *HSPB7* relative promoter methylation in eighteen tumor types and their matched normal tissues. Promoter methylation ratios of tumor to normal for *SRARP* and *HSPB7* genes were calculated in each tumor type, and statistical significance analysis was carried out in each dataset using a *t*‐test. *P < 0.005. Green and red colors denote decrease and increase in relative promoter methylation, respectively. (C–D) The associations between the promoter methylation and expression values for *SRARP* were measured by Pearson correlation coefficient (CC) and linear regression curve estimation in breast (C) and prostate (D) cancers.

Comparing gene expression and promoter methylation between tumors and their matched normal tissues are informative in understanding the role of cancer genes in the process of malignant transformation. Therefore, *SRARP* and *HSPB7* gene expression and promoter methylation were analyzed in eighteen tumor types and their respective normal tissues using TCGA datasets as explained in methods. It is notable that the source of normal samples in the majority of TCGA datasets was histologically normal tissues adjacent to tumors. Gene expression data were derived from RNA‐seq RPKM values in TCGA Data Portal using MethHC 1.0.3. Median *SRARP* and *HSPB7* expression levels were obtained for tumor and normal samples in each dataset and differential gene expression values were calculated as follows: log2 (RPKM+1)‐transformed median values of tumor ‐ log2 (RPKM+1) of normal. Next, *P* values for differential expression between tumor and normal pairs were calculated using the Mann–Whitney *U*‐test (Table 2 and Fig. 3A).

**Table 2:** Gene expression of *SRARP* and *HSPB7* in tumors and normal tissues and correlations between expression and promoter methylation. Gene expression values were obtained from RNA‐seq RPKM (reads per kilobase per million mapped reads) values in TCGA Data Portal using MethHC 1.0.3. Median expression levels are shown for tumor (T) and normal (N) samples. *P* values for differential expression (Diff. exp.) between T and N were calculated using the Mann–Whitney *U*‐test. The associations between the promoter methylation and gene expression (Met‐Exp.) were measured by Pearson correlation coefficient (CC)

	RPKM (T)	RPKM (N)	*P* (Diff. exp.)	No. (T)	No. (N)	CC (Met‐Exp.)	*P* (CC)
Tissue (SRARP)							
blca	0	0.4	>0.1	241	19	−0.07	>0.1
brca	234	180	0.027*	1041	109	−0.6	<0.001*
cesc	0	5.3	0.055	185	3	−0.21	0.005*
coad	0	0	>0.1	262	29	−0.13	0.043*
hnsc	0	1.3	<0.001*	497	42	−0.04	>0.1
kirc	0.3	34	<0.001*	518	72	−0.55	<0.001*
kirp	0.62	51	<0.001*	172	30	−0.37	<0.001*
lihc	0.59	0.63	>0.1	191	50	−0.53	<0.001*
luad	0.28	0.56	>0.1	488	42	−0.27	0.007*
lusc	0	1	<0.001*	409	46	−0.02	>0.1
paad	0	0	>0.1	85	3	−0.2	0.065
prad	53	21	<0.001*	297	50	−0.66	<0.001*
read	0	0.55	0.017*	91	6	−0.17	>0.1
sarc	0	43.34	0.004*	103	2	0.01	>0.1
skcm	0	0	>0.1	372	1	−0.15	0.003*
stad	0	0.04	0.008*	285	33	−0.45	<0.001*
thca	0.71	37.27	<0.001*	498	59	−0.4	<0.001*
ucec	1.88	1.09	>0.1	159	12	−0.43	<0.001*
Tissue (HSPB7)							
blca	33	1103	<0.001*	241	19	0.04	>0.1
brca	77	2526	<0.001*	1041	109	−0.07	0.06
cesc	35	2652	0.003*	185	3	−0.14	>0.1
coad	47	354	<0.001*	282	28	−0.15	>0.1
hnsc	28	324	<0.001*	497	42	0.04	>0.1
kirc	32	434	<0.001*	518	72	−0.43	<0.001*
kirp	39	722	<0.001*	172	30	−0.15	0.072
lihc	11	22	0.008*	191	50	−0.25	0.001*
luad	66	235	<0.001*	488	58	−0.16	0.001*
lusc	32	328	<0.001*	409	50	−0.07	>0.1
paad	145	120	>0.1	85	3	−0.13	>0.1
prad	251	683	<0.001*	297	50	−0.12	0.042*
read	187	947	0.002*	91	6	0.22	>0.1
sarc	607	3806	>0.1	103	2	−0.52	<0.001*
skcm	15	6	>0.1	372	1	−0.1	0.055
stad	2	9	<0.001*	285	33	−0.32	<0.001*
thca	101	229	<0.001*	498	59	−0.05	>0.1
ucec	22	1830	<0.001*	159	12	−0.26	0.001*

blca, bladder cancer (CA); brca, breast CA; cesc, cervical CA; coad, colon CA; hnsc, head and neck CA; kirc, renal clear cell CA; kirp, renal papillary CA; lihc, liver CA; luad, lung adeno‐CA; lusc, lung squamous CA; No., number; paad, pancreatic CA; prad, prostate CA; read, rectal CA; sarc, sarcoma; skcm, skin melanoma; stad, stomach CA; thca, thyroid CA; ucec, endometrial CA.

*Depicts significance at *P* < 0.05.

Steroid receptor associated and regulated protein expression showed a significant increase in breast and prostate cancers compared to their matched normal tissues with differential expression values of 0.4 (*P* = 0.027) and 1.29 (*P* < 0.001), respectively (Fig. 3A and Table 2). In contrast, *SRARP* differential expression showed significant negative values between −0.06 and −5.47 (*P* < 0.02) in head and neck, renal clear cell, renal papillary, lung squamous cell, rectal, stomach, and thyroid cancers in addition to sarcoma (Fig. 3A and Table 2). However, *SRARP* expression did not show a significant change in the remaining eight tumor types compared to their matched normal tissues (Fig. 3A). In addition, *HSPB7* differential expression demonstrated negative values between −0.94 and −6.32 (*P* < 0.01) in fifteen tumor types, namely bladder, breast, cervical, colon, head and neck, renal clear cell, renal papillary, liver, lung squamous cell, prostate, rectal, stomach, thyroid, and endometrial cancers in addition to lung adenocarcinoma (Fig. 3A and Table 2). There was no significant change in *HSPB7* differential expression in the remaining three cancers (Fig. 3A). These findings suggest that *SRARP* and *HSPB7* expression levels are significantly reduced in multiple malignancies compared to their normal tissues; however, *SRARP* expression is relatively increased in breast and prostate cancers.

Furthermore, promoter methylation analysis in eighteen tumor types and their matched normal tissues were carried out using MethHC on the data obtained from TCGA Data Portal as explained in methods. Next, promoter methylation ratios of tumor to normal for *SRARP* and *HSPB7* genes were calculated in each tumor type and statistical significance was tested for the differences between tumor and normal samples in each dataset using a *t*‐test. Subsequently, the associations between the promoter methylation and expression values for *SRARP* and *HSPB7* in eighteen tumor datasets were measured by PCC and linear regression curve estimation.

Of note, breast cancer showed the most reduction in the *SRARP* promoter methylation compared to its matched normal tissue with a relative promoter methylation of 0.78‐fold (*P* < 0.005; Fig. 3B and Table S3). In addition, there was a significant decrease in the relative promoter methylation of *SRARP* between 0.89‐ and 0.95‐fold in colon, liver, prostate, rectal, and endometrial cancers (*P* < 0.005; Fig. 3B and Table S3). In contrast, *SRARP* promoter methylation was significantly increased in cervical, head and neck, renal clear cell, renal papillary and thyroid cancers in addition to sarcoma and skin melanoma by 1.05‐ to 1.25‐fold (*P* < 0.005; Fig. 3B and Table S3). Furthermore, *HSPB7* relative promoter methylation was significantly increased in nine cancer types by 1.07‐ to 1.37‐fold and reduced only in lung squamous cell and thyroid cancers by 0.9‐ and 0.83‐fold, respectively (*P* < 0.005; Fig. 3B and Table S3).

Moreover, *SRARP* expression and promoter methylation had a significant inverse correlation in twelve of eighteen TCGA datasets (*P* < 0.05; Table 2). Importantly, the two strongest inverse correlations were detected in prostate and breast cancers with PCC values of −0.66 and −0.6, respectively (*P* < 0.001; Fig. 3C,D and Table 2). In addition, gene expression and promoter methylation of *HSPB7* showed a significant inverse correlation in seven tumor types (*P* < 0.05; Table 2). Therefore, the promoter methylation levels of *SRARP* and *HSPB7* are significantly altered in multiple cancer types compared to their matched normal tissues, showing hypermethylation in the majority of changes. Of note, *SRARP* promoter methylation and gene expression inversely correlate in most tumor types, and particularly promoter hypomethylation is associated with the observed increase in *SRARP* expression in breast and prostate cancers.

To investigate the regulation of SRARP and HSPB7 expression in malignancies, a broad group of cancer cell lines were applied that included breast cancer lines T‐47D (ER+/AR+), MFM‐223 (ER−/AR+), MDA‐MB‐231 (ER−/AR−), and MDA‐MB‐468 (ER−/AR−); prostate cancer line DU‐145 (AR‐independent); renal carcinoma lines 786‐0 and A498; UACC‐257 (melanoma); U‐2 OS (osteosarcoma); IGROV1 (ovarian cancer); Ishikawa (endometrial cancer); A549 (non‐small‐cell lung cancer); HCT‐15 (colorectal cancer); and GBM line SF‐268 (Table 3). It is notable that heat shock induction and chemical induced hypoxia using CoCl2 treatment did not increase the expression of *SRARP* and *HSPB7* in T‐47D and MFM‐223 cells, indicating that they are not involved in the transcriptional regulation of these genes (Fig. S1).

**Table 3:** Expression of *HSPB7* and *SRARP* in fourteen cancer cell lines. −ΔCT is −Δ cycle threshold value (±standard error of the mean) for *HSPB7* and *SRARP* expression using qRT‐PCR. −ΔCT value is proportional to the amount of target mRNA in the sample. Name of cell lines and their cancer types are listed. AR, androgen receptor; ER, estrogen receptor; GBM, glioblastoma multiforme; NSC, non‐small‐cell. Experiments were performed in four replicates

Cell line	Cancer type	−ΔCT *HSPB7*	−ΔCT *SRARP*
T‐47D	Breast (ER+/AR+)	−17.86 (±0.3)	−7.24 (±0.04)
MFM‐223	Breast (ER−/AR+)	−17.72 (±0.3)	−4.67 (±0.02)
786‐0	Renal	−13.75 (±0.1)	−20.20 (±0)
A498	Renal	−13.84 (±0.3)	−19.32 (±0)
DU‐145	Prostate (AR‐independent)	−17.16 (±0.4)	−20.93 (±0)
UACC‐257	Melanoma	−20.34 (±0.74)	−17.48 (±0.37)
U‐2 OS	Osteosarcoma	−8.37 (±0.24)	−20.97 (±0)
MDA‐MB‐231	Breast (ER−/AR−)	−16.58 (±0.55)	−21.06 (±0)
MDA‐MB‐468	Breast (ER−/AR−)	−14.72 (±0.22)	−14.69 (±0.17)
IGROV1	Ovarian	−17.20 (±0.21)	−20.84 (±0.4)
Ishikawa	Endometrial	−16.21 (±0.36)	−17.87 (±0.53)
A549	Lung (NSC)	−17.32 (±0.16)	−14.69 (±0.39)
HCT‐15	Colorectal	−14.39 (±0.33)	−21.27 (±0)
SF‐268	Brain (GBM)	−17.05 (±0.71)	−21.24 (±0)

Next, the effects of DNA methylation and histone deacetylation on the epigenetic regulation of *SRARP* and *HSPB7* were examined using demethylation and HDAC inhibition with 5‐aza‐dC and TSA, respectively. A total of fourteen cancer cell lines were treated with 5‐aza‐dC and TSA followed by the assessment of *SRARP* and *HSPB7* expression using qRT‐PCR. Fold change in gene expression was calculated in each cell line as gene expression in the treated group/average gene expression in the control group. Examination of the baseline gene expression revealed that *SRARP* is highly expressed only in T‐47D and MFM‐223 cell lines with −ΔCT (−Δ cycle threshold) values of −7.24 (±0.04) and −4.67 (±0.02), respectively (Table 3). It is notable that −ΔCT value is proportional to the amount of target mRNA in the sample (Childs *et al*., 2009; Kawarazaki *et al*., 2010). These *SRARP* transcript levels are in agreement with the high levels of SRARP protein detected in T‐47D and MFM‐223 cell lines (Naderi, 2017). In contrast, baseline expression levels of *SRARP* were low in the remaining twelve cancer cell lines, showing −ΔCT values between −14.69 and −21.27 (Table 3). Similarly, *HSPB7* demonstrated low baseline expression levels in thirteen cancer cell lines with −ΔCT values measuring from −13.75 to −20.34 (Table 3). However, osteosarcoma cell line U‐2 OS showed a relatively higher baseline *HSPB7* expression at a −ΔCT of −8.37 (±0.24) (Table 3).

**Figure 4:**
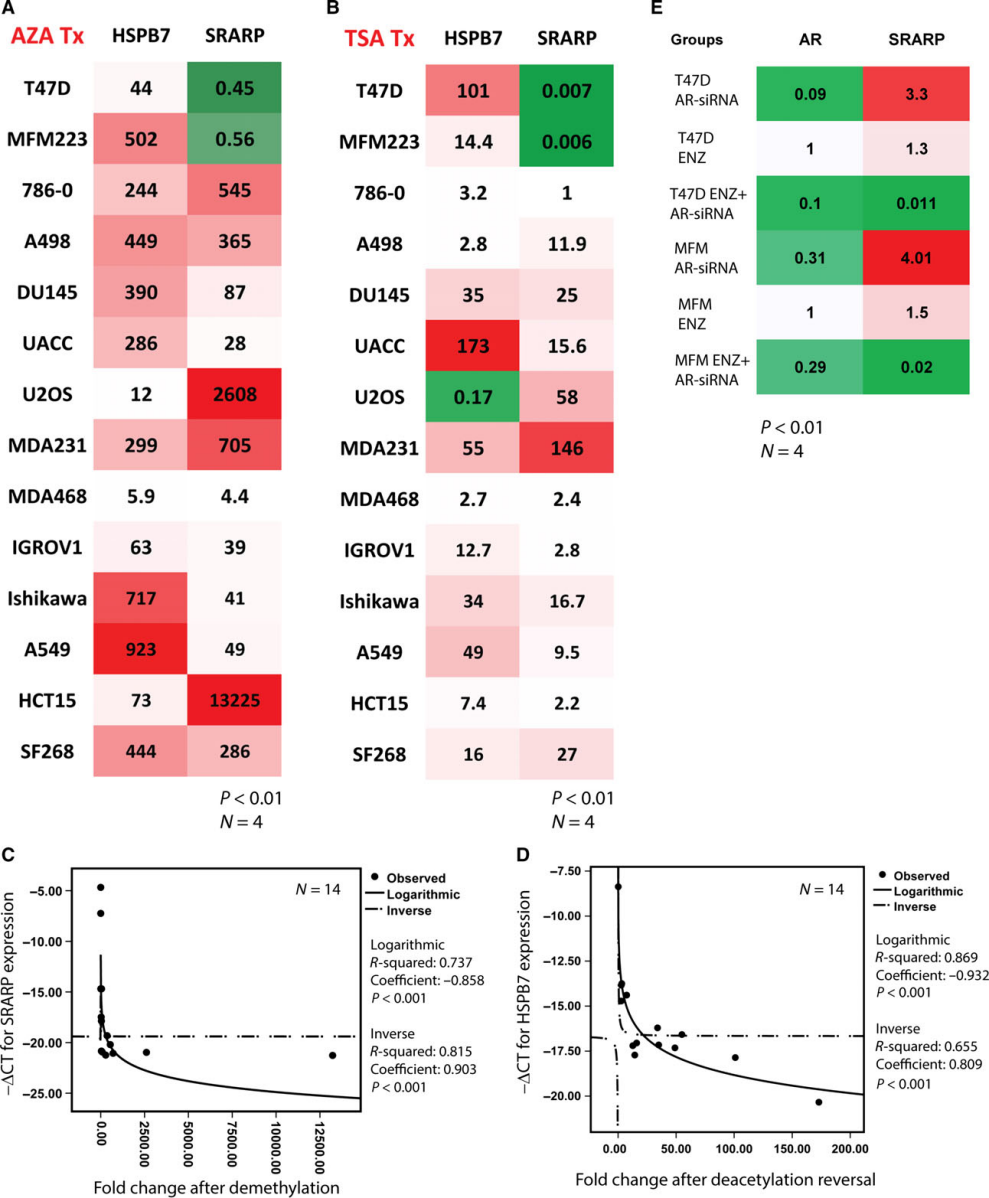
Gene expression data using qRT‐PCR for the epigenetic regulation of *SRARP* and *HSPB7* in cancer cells and the effect of AR inactivation on SRARP. (A) Heat map showing relative expression of *SRARP* and *HSPB7* following DNA demethylation using AZA in cancer cell lines. *P* value is the significance of fold change between AZA‐treated and control cells using a *t*‐test. All fold changes are significant at a *P* < 0.01. MDA231: MDA‐MB‐231; MDA468: MDA‐MB‐468. Green and red colors denote decrease and increase in expression, respectively. (B) Heat map showing relative expression following the HDAC inhibition using TSA in cancer cell lines. *P* value is for the significance of fold change between TSA‐treated and control cells using a *t*‐test. Fold changes ≠ 1 are significant at a *P* < 0.01. (C and D) Regression models to predict *SRARP* and *HSPB7* expression based on their epigenetic regulation in cancer cell lines. (C) Logarithmic and inverse regression models to predict *SRARP* expression after DNA demethylation using AZA. (D) Logarithmic and inverse regression models to predict *HSPB7* expression after HDAC inhibition using TSA. −ΔCT is −Δ cycle threshold value for gene expression. R‐squared values, standardized coefficients, and *P* values are shown. (E) Heat map to show fold changes in *AR* and *SRARP* expression using qRT‐PCR after AR silencing (AR‐siRNA), enzalutamide (ENZ) treatment, or combined ENZ and AR‐siRNA in T‐47D and MFM‐223 cell lines. Fold changes ≠ 1 are significant at a *P* < 0.01. Green and red colors denote decrease and increase in expression, respectively.

Following the induction of demethylation in cancer cell lines using 5‐aza‐dC, there was a significant increase in *SRARP* expression in twelve cell lines by 4.4‐ to 13 225‐fold (*P* < 0.01; Fig. 4A). Conversely, *SRARP* was reduced following demethylation by approximately twofold in T‐47D and MFM‐233 cell lines (*P* < 0.01; Fig. 4A). In addition, *HSPB7* expression was significantly increased following 5‐aza‐dC treatment in all fourteen cancer cell lines by 5.9‐ to 923‐fold (*P* < 0.01; Fig. 4A). Importantly, histone deacetylation reversal using TSA treatment produced a similar effect on *SRARP* expression to that observed with demethylation (Fig. 4B). In this respect, *SRARP* expression was significantly increased in eleven cancer lines by 2.2‐ to 146‐fold (*P* < 0.01; Fig. 4B). In contrast, *SRARP* transcription was markedly reduced following TSA in T‐47D and MFM‐223 cells by more than 100‐fold (*P* < 0.01; Fig. 4B). Furthermore, *HSPB7* expression was significantly increased following HDAC inhibition in thirteen cancer cell lines by 2.7‐ to 173‐fold (*P* < 0.01; Fig. 4B). However, U‐2 OS cell line, which has a relatively higher baseline expression of *HSPB7*, showed a significant reduction in *HSPB7* transcription following TSA by approximately fivefold (*P* < 0.01; Fig. 4B). These findings suggest that *SRARP* expression and *HSPB7* expression are silenced by methylation and histone deacetylation in cancer cell lines of multiple tissue origins.

Moreover, statistical modeling using regression analysis was performed to predict *SRARP* and *HSPB7* expression based on their epigenetic regulation data by DNA demethylation and histone deacetylation reversal in fourteen cancer cell lines. Notably, *SRARP* expression was highly predicable using logarithmic and inverse regression models generated based on the demethylation and HDAC inhibition results (Figs 4C and S2A). In this respect, demethylation data predicted *SRARP* expression using logarithmic and inverse regression models with *R*‐squared values of 0.737 (*P* < 0.001, coefficient: −0.858) and 0.815 (*P* < 0.001, coefficient: 0.903), respectively (Fig. 4C). Correspondingly, HDAC inhibition results also predicted *SRARP* expression using logarithmic and inverse regression models with R–squared values of 0.723 (*P* < 0.001, coefficient: −0.850) and 0.819 (*P* < 0.001, coefficient: 0.905), respectively (Fig. S2A). However, *HSPB7* expression was predicable only based on HDAC inhibition data, showing *R*‐squared values of 0.869 (*P* < 0.001, coefficient: −0.932) and 0.655 (*P* < 0.001, coefficient: 0.809) for logarithmic and inverse regression models, respectively (Figs 4D and S2B).

Collectively, these findings strongly suggest epigenetic silencing as a key factor in the regulation of *SRARP* and *HSPB7* expression across tumors and cancer cell lines of multiple tissue origins. In this respect, *SRARP* is hypermethylated in multiple malignancies and its expression inversely correlates with the promoter methylation levels in tumors. Importantly, in breast and prostate cancers, a relative increase in *SRARP* expression in tumors is associated with the hypomethylation of its promoter. In addition, *SRARP* shows DNA hypermethylation and histone deacetylation in most cancer cell lines. Interestingly, in T‐47D and MFM‐223 lines that have a high baseline level of *SRARP*, demethylation and HDAC inhibition lead to a reduction in gene expression, indicating that it is not epigenetically silenced in these cells. Moreover, *HSPB7* shows DNA hypermethylation in most tumors and all tested cell lines, and has histone deacetylation in most cancer cells. Finally, the effect of epigenetic silencing by DNA methylation and/or histone deacetylation strongly predicts *SRARP* and *HSPB7* expression across multiple cancer cell lines.

### 3.3 AR has dual regulatory effects on *SRARP* transcription

Androgen receptor and SRARP are highly co‐expressed in breast cancer and there are high levels of SRARP expression in AR+ breast cancer cell lines T‐47D and MFM‐223 (Table 3) (Naderi, 2017). In contrast, AR‐ breast cancer lines MDA‐MB‐231 and MDA‐MB‐468 have low expression levels of *SRARP* (Table 3). Furthermore, it is known that AR activation directly suppresses *SRARP* transcription in MFM‐223 and T‐47D cell lines (Naderi, 2017). Collectively, these findings raise the question whether a minimum level of AR activity may be required for baseline expression of *SRARP* in AR+ cancer cells, while higher levels of AR activity suppress this gene. This possibility was examined in T‐47D and MFM‐223 cell lines following AR‐siRNA silencing, AR inhibition with enzalutamide treatment at 10 μm concentration, and a combination of AR silencing and enzalutamide treatment. Experiments were performed over 72 h in four replicates and *SRARP* expression was measured in each group relative to that of control siRNA using qRT‐PCR.

Androgen receptor silencing reduced *AR* expression by approximately 90% and 70% in T‐47D and MFM‐223 cells, respectively (*P* < 0.01; Fig. 4E). Consistent with author's published data (Naderi, 2017), AR silencing alone significantly increased *SRARP* expression by 3.3‐ and 4‐fold in T‐47D and MFM‐223 cell lines, respectively (*P* < 0.01; Fig. 4E). In addition, enzalutamide treatment moderately increased *SRARP* expression by 1.3‐ and 1.5‐fold in T‐47D and MFM‐223 cell lines, respectively (*P* < 0.01; Fig. 4E). Conversely, the combination of AR silencing and enzalutamide treatment markedly reduced *SRARP* expression by 86‐ and 53‐fold in T‐47D and MFM‐233 cells, respectively (*P* < 0.01; Fig. 4E). These findings suggest that AR exerts dual regulatory effects on *SRARP* expression and although an increased AR activity suppresses *SRARP* transcription, a minimum level of AR activity is required to maintain baseline *SRARP* expression in AR+ breast cancer cells.

### 3.4 *SRARP* and *HSPB7* genes are commonly deleted in malignancies

**Figure 5:**
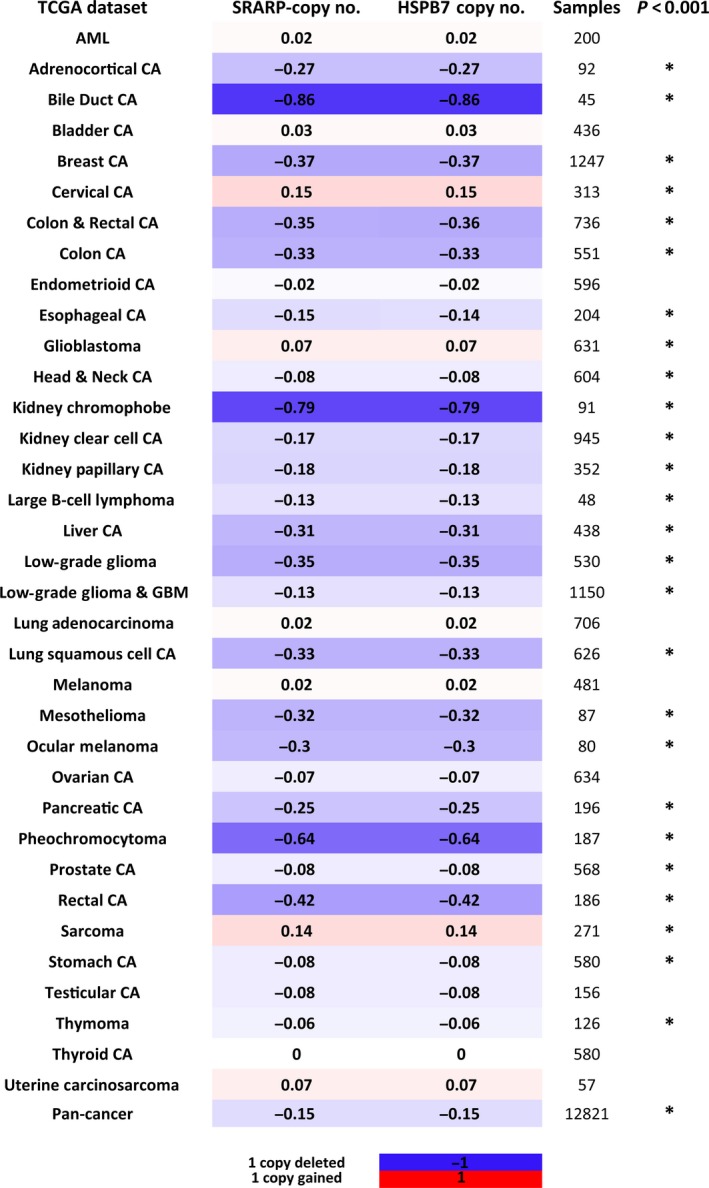
Steroid receptor associated and regulated protein and HSPB7 copy number changes across different malignancies. Heat map demonstrates mean gene‐level estimates for *SRARP* and *HSPB7* in 35 TCGA datasets and TCGA Pan‐Cancer dataset. The GISTIC2_thersholded method was utilized to measure *SRARP* and *HSPB7* gene‐level copy number changes. Next, significance levels for copy number changes between cancer types and their matched normal tissues were calculated using the Kruskal–Wallis test. For each TCGA dataset, the name of dataset, *SRARP* and *HSPB7* copy number changes (copy no.), and sample size are shown. *P < 0.001 for copy no. Blue and red colors denote decease and increase in gene‐level copy numbers, respectively.

It is notable that deletions involving chromosome 1p in general and 1p36 in particular commonly occur in cancer (Henrich *et al*., 2012; Knuutila *et al*., 1999). In view of the fact that *SRARP* and *HSPB7* genes are located on the 1p36 region, the possibility of their gene‐level changes was investigated in malignancies. To achieve this, *SRARP* and *HSPB7* copy number variations were examined in cancers compared to their matched normal tissues. A total of 35 TCGA datasets across different malignancies in addition to TCGA Pan‐Cancer dataset were analyzed as explained in methods. The GISTIC2_thersholded method was utilized to measure *SRARP* and *HSPB7* gene‐level copy number changes. Next, significance levels for copy number changes between cancers and their matched normal tissues were calculated using the Kruskal–Wallis test and mean gene‐level estimates were applied to create a heat map (Fig. 5).

Notably, *SRARP* and *HSPB7* genes had significant deletions in 23 of 35 TCGA datasets ranging from −0.06 to −0.86 copies (*P* < 0.001; Fig. 5). The two highest copy number losses were observed in bile duct and kidney chromophobe cancers, showing a loss of −0.86 and −0.79 copies, respectively (*P* < 0.001; Fig. 5). As expected from being gene pairs, *SRARP* and *HSPB7* had an identical pattern of copy number changes across different malignancies (Fig. 5). In addition, *SRARP* and *HSPB7* showed significant copy number gains in only three cancer types, namely cervical cancer, sarcoma, and glioblastoma with gains of 0.15, 0.14, and 0.07 copies, respectively (*P* < 0.001; Fig. 5). Importantly, analysis of TCGA Pan‐Cancer dataset demonstrated that *SRARP* and *HSPB7* have an average loss of −0.15 copies across a total of 12 821 malignant samples (*P* < 0.001; Fig. 5). Therefore, *SRARP* and *HSPB7* genes are widely deleted in malignancies of multiple tissue origins.

### 3.5 SRARP and HSPB7 function as tumor suppressors

The combination of epigenetic silencing and gene‐level deletions of *SRARP* and *HSPB7* across multiple malignancies raised the question whether these genes have a tumor suppressor function. To investigate this possibility, clonogenic assays were carried out to assess colony formation in stably transfected cancer cells derived from different tissue origins. MDA‐MB‐231 (breast cancer), DU‐145 (prostate cancer), and A549 (non‐small‐cell lung cancer) cell lines were employed for colony forming assays in view of the fact that they all have low levels of *SRARP* and *HSPB7* expression (Table 3). Cell lines were transfected with each cDNA clone containing *SRARP* (SRARP+), *HSPB7* (HSPB7 + ) or an empty plasmid (CTL‐VEC). Forty‐eight hours following transfections, a total of 1000 cells were seeded in 6‐well plates and cultured for 21 days in selection medium to generate stable lines. Plates were then stained with 0.5% crystal violet and colonies containing more than 50 cells were counted. The overexpression of SRARP and HSPB7 proteins were confirmed by western blotting 48 h after transfection of constructs.

**Figure 6:**
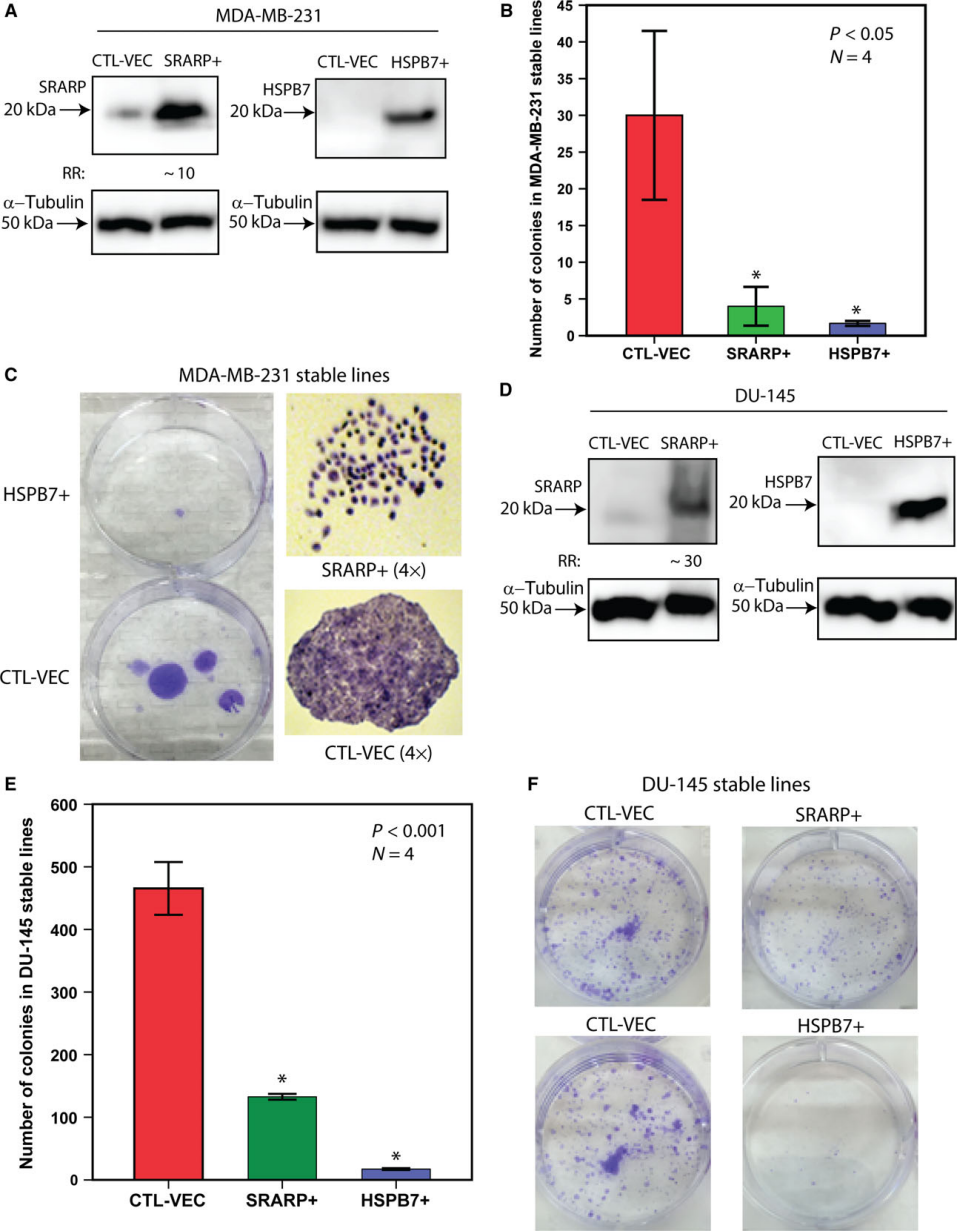
Effects of SRARP and HSPB7 overexpression on colony formation. (A) Western blotting to assess SRARP and HSPB7 overexpression in MDA‐MB‐231 cells following transfections. Fold change (RR) in each band density was measured relative to control in three replicate experiments. CTL‐VEC: control vector; SRARP+: SRARP overexpression; HSPB7 + : HSPB7 overexpression. (B) Clonogenic assays to assess colony formation in stably transfected MDA‐MB‐231 cells. A total of 1000 cells containing *SRARP* (SRARP+), *HSPB7* (HSPB7 + ), or CTL‐VEC were cultured for 21 days in selection medium, and colonies with more than 50 cells were counted. Experiments were carried out in four replicates. ANOVA with Dunnett's *post hoc* test was applied to calculate the statistical significance. *P < 0.05 for SRARP+ or HSPB7 +  vs. CTL‐VEC. Error bars depict ± SEM. (C) Representative images of plates containing CTL‐VEC and HSPB7 +  MDA‐MB‐231 lines and microscopic images of representative clones from SRARP+ and CTL‐VEC lines (4X). (D) Western blotting to assess SRARP and HSPB7 overexpression in DU‐145 cells as explained in (A). (E) Clonogenic assays to assess colony formation in stably transfected DU‐145 cells as described in (B). *P < 0.001 for SRARP+ or HSPB7+ vs. CTL‐VEC. (F) Representative images of plates containing CTL‐VEC, SRARP+, and HSPB7+ DU‐145 lines.

In MDA‐MB‐231 cell line, SRARP and HSPB7 protein overexpression was confirmed in SRARP+ and HSPB7+ cells, respectively (Fig. 6A). SRARP showed a low level of protein expression in CTL‐VEC cells that was increased by 10‐fold following SRARP overexpression (Fig. 6A). In addition, HSPB7 protein was not detectable in CTL‐VEC cells but there was a distinct protein band in HSPB7 +  cells (Fig. 6A). Importantly, SRARP+ and HSPB7+ MDA‐MB‐231 stable lines demonstrated a marked reduction in the number of colonies compared to that of CTL‐VEC stable line by 6.5‐ and 15‐fold, respectively (*P* < 0.05; Fig. 6B,C). It is notable that colonies were both visibly and microscopically smaller in SRARP+ and HSPB7+ stable lines compared to those of CTL‐VEC line (Fig. 6C).

**Figure 7:**
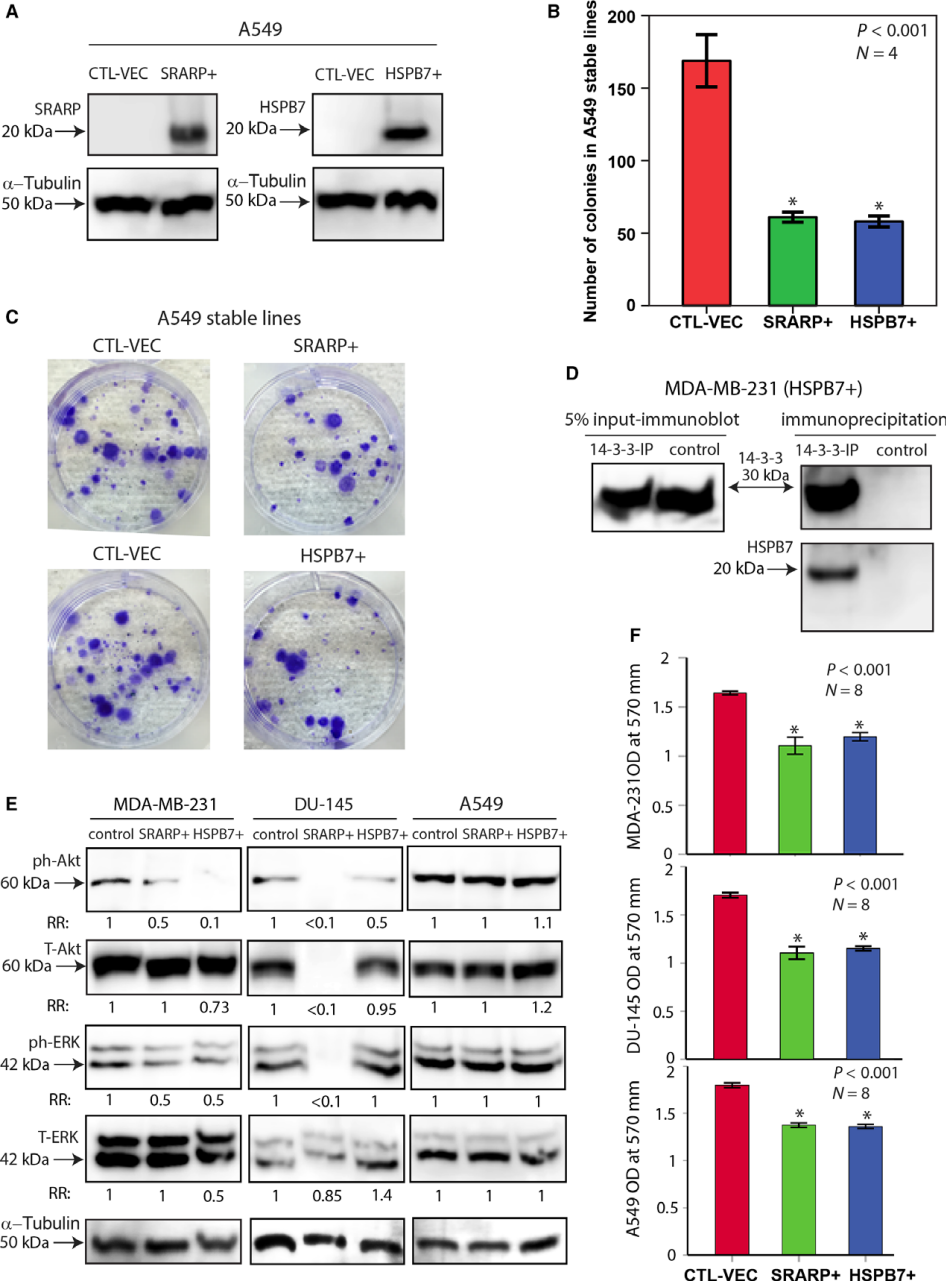
Effects of SRARP and HSPB7 overexpression on colony formation and signaling pathways. (A) Western blotting to assess SRARP and HSPB7 overexpression in A549 cells following transfections. CTL‐VEC: control vector; SRARP+: SRARP overexpression; HSPB7+: HSPB7 overexpression. (B) Clonogenic assays in stably transfected A549 cells containing SRARP (SRARP+), HSPB7 (HSPB7+), or CTL‐VEC. Experiments were carried out in four replicates. ANOVA with Dunnett's *post hoc* test was applied to calculate the statistical significance. *P < 0.001 for SRARP+ or HSPB7+ vs. CTL‐VEC. Error bars depict ± SEM. (C) Representative images of plates containing CTL‐VEC, SRARP+, and HSPB7+ A549 lines. (D) Co‐immunoprecipitation to examine the interaction between 14‐3‐3 and HSPB7 in HSPB7‐transfected MDA‐MB‐231 cells. IP assay was performed using a 14‐3‐3 antibody, and control experiment was conducted with a nonspecific rabbit IgG. Western blotting on IP lysates was carried out using 14‐3‐3 and HSPB7 antibodies, and input was assessed by 14‐3‐3 immunoblotting. (E) Western blot analysis in MDA‐MB‐231, DU‐145, and A549 cell lines following transfections with SRARP (SRARP+), HSPB7 (HSPB7+), and CTL‐VEC (control). Protein levels for phospho‐Akt (ph‐Akt), total Akt (T‐Akt), phospho‐ERK (ph‐ERK), and total ERK (T‐ERK) were assessed 48 h after transfections. Each fold change (RR) is the average band density measured relative to its respective control across three replicates. (F) MTT assays to measure cell viability in MDA‐MB‐231, DU‐145, and A549 cell lines stably transfected with SRARP (SRARP+), HSPB7 (HSPB7+), or CTL‐VEC. OD at 570 mm is measured at 72‐h time point in each line. *P < 0.001 for SRARP+ or HSPB7+ vs. CTL‐VEC. Error bars depict ± SEM.

Furthermore, in DU‐145 cells, western blotting confirmed SRARP and HSPB7 overexpression following transfections compared to control cells (Fig. 6D). SRARP had a faint protein band in CTL‐VEC‐transfected cells that was increased by 30‐fold in SRARP+ cells (Fig. 6D). HSPB7 protein was not detectable in CTL‐VEC; however, it had a strong expression in HSPB7+ cells (Fig. 6D). Of note, SRARP+ and HSPB7+ DU‐145 stable lines developed significantly less colonies compared to CTL‐VEC line by 3.5‐ and 27‐fold, respectively (*P* < 0.001; Fig. 6E,F). Moreover, in A549 cells, SRARP and HSPB7 overexpression was confirmed by the presence of strong protein bands in SRARP+ and HSPB7+ lines, respectively (Fig. 7A). In contrast, SRARP and HSPB7 proteins were not detectable in CTL‐VEC A549 cells (Fig. 7A). In addition, colony numbers were significantly reduced in SRARP+ and HSPB7+ A549 stable lines compared to that of CTL‐VEC line by 2.7‐ and 3‐fold, respectively (*P* < 0.001; Fig. 7B,C). Therefore, SRARP or HSPB7 overexpression in cancer cell lines leads to a marked reduction in clonogenicity, suggesting that these proteins function as tumor suppressors.

In view of the fact that *SRARP* and *HSPB7* are co‐expressed gene pairs with tumor suppressor functions, the possibility of similarities between the molecular features of these proteins was further investigated. It is notable that a biochemical feature of SRARP is an interaction with the endogenous 14‐3‐3 protein (Naderi, 2017). In addition, bioinformatics analysis predicted that there may be a similar interaction between HSPB7 and 14‐3‐3 proteins (Fig. 2B). Therefore, co‐IP assay was performed in HSPB7‐transfected MDA‐MB‐231 cells to examine whether 14‐3‐3 and HSPB7 are binding partners. IP experiments were conducted using a 14‐3‐3 antibody and a nonspecific rabbit IgG was applied for control. Next, western blotting was carried out on IP lysates using 14‐3‐3 and HSPB7 antibodies. Furthermore, 5% of lysate was collected before IP to assess input by western blot using a 14‐3‐3 antibody (Fig. 7D). Notably, immunoblotting with a 14‐3‐3 antibody confirmed the successful IP of 14‐3‐3 protein (Fig. 7D). Furthermore, HSPB7 antibody detected a distinct protein band for HSPB7 in the 14‐3‐3 IP assay, which was absent in the control IP (Fig. 7D). These findings indicate that HSPB7 interacts with the 14‐3‐3 protein.

Next, the signaling effects of SRARP and HSPB7 overexpression were assessed by measuring the protein levels of phospho‐Akt (ph‐Akt), total Akt (T‐Akt), phospho‐ERK (ph‐ERK), and total ERK (T‐ERK). In this respect, MDA‐MB‐231, DU‐145, and A549 cell lines were transfected with each of the *SRARP* (SRARP+), *HSPB7* (HSPB7+), and CTL‐VEC (control) plasmids and protein lysates were harvested 48 h following transfections. Western blot analysis was carried out to detect the level of proteins and fold change (RR) in each band density was measured relative to its respective control in three replicate experiments. Finally, the average RR for each protein was obtained across replicates (Fig. 7E).

Notably, SRARP overexpression led to a reduction in the relative Akt phosphorylation (ph‐Akt/T‐Akt) by twofold in MDA‐MB‐231 cells and a marked decrease in T‐Akt expression by over 10‐fold in DU‐145 cells accompanied by a corresponding reduction in ph‐Akt (Fig. 7E). In addition, SRARP+ cells demonstrated a reduction in ph‐ERK/T‐ERK ratio by 2‐ and 10‐fold in MDA‐MB‐231 and DU‐145 cell lines, respectively (Fig. 7E). Furthermore, HSPB7 overexpression decreased the relative Akt phosphorylation by approximately eight‐ and twofold in MDA‐MB‐231 and DU‐145 cells, respectively (Fig. 7E). Moreover, HSPB7+ MDA‐MB‐231 cells showed a twofold reduction in ph‐ERK and T‐ERK levels compared to control (Fig. 7E). However, there was no measurable change in Akt and ERK protein levels in SRARP+ and HSPB7+ A549 cells (Fig. 7E). These findings suggest that SRARP and HSPB7 overexpression may reduce the relative phosphorylation and/or expression of Akt and ERK proteins in cancer cells.

The effects of SRARP and HSPB7 overexpression on cell viability were assessed in MDA‐MB‐231, DU‐145, and A549 cell lines using MTT assay. Stably transfected SRARP+ and HSPB7+ cell lines were seeded at 5.000 cells per well in a 96‐well plate and cultured for 72 h followed by MTT assay. Stable transfections of an empty plasmid were used as controls. There was a significant reduction in cell viability in SRARP+ and HSPB7+ MDA‐MB‐231 and DU‐145 cells by 30 to 35% and in A549 cells by 25% compared to control cells over a 72‐h time period (*P* < 0.001; Fig. 7F). Therefore, SRARP or HSPB7 overexpression significantly reduces cell viability in cancer cell lines.

Moreover, functional annotation of *SRARP*‐signature genes was examined in a cohort of 50 breast cancer cell lines as explained in methods. In this respect, two signatures were identified for positively and inversely correlated genes with *SRARP* expression at PCC cutoffs of ≥ 0.6 and ≤ −0.6, respectively (*P* < 0.001; Table S4). Next, functional annotation clustering of each signature was carried out using DAVID Bioinformatics Resources. Of note, positively and inversely correlated genes demonstrated opposite annotation terms related to the tumorigenic functions (Tables 4 and S5). In particular, positively correlated signature was associated with the negative regulation of signal transduction, while inversely correlated signature was enriched for genes related to the positive regulation of signal transduction, cell proliferation, protein kinase activity, and phosphorylation (Table 4). Inversely correlated genes were also enriched for SH3 domain, Notch signaling, and integrin binding (Table 4). In addition, positively correlated genes were associated with hormone stimulus, adaptor, and transcription factor activity (Table 4). Therefore, *SRARP* is inversely correlated with the expression of genes that promote cancer cell growth and signal transduction in support of its function as a tumor suppressor.

**Table 4:** Summary of functional annotation clustering for positively and inversely correlated *SRARP*‐signature genes obtained in 50 breast cancer cell lines. Positively and inversely correlated genes have Pearson correlation coefficients of ≥ 0.6 and ≤ −0.6 with *SRARP* expression, respectively. Functional annotation clustering was conducted using DAVID Bioinformatics Resources at a significance level of *P* < 0.05

Terms for positively correlated genes	Terms for inversely correlated genes
Cytoplasmic vesicle, transmembrane	SH3 domain, Src homology
Ion transport, ion binding	Lipid transport, lipid moiety‐binding
Response to hormone stimulus	Notch signaling pathway
Adaptor activity, intracellular transport	Cell motion, positive regulation of cell proliferation
Negative regulation of signal transduction	Positive regulation of signal transduction
GTPase and ATPase regulator activities	Positive regulation of protein kinase activity
Mammary gland development	Regulation of phosphorylation
Golgi apparatus	Focal adhesion and integrin binding
Transcription factor activity	Regulation of protein polymerization and metabolic process
Chemical homeostasis	Immune response

To further investigate SRARP‐associated pathways, gene sets that are co‐expressed with *SRARP* at a CC> 0.6 were identified in breast and prostate cancers using the average linkage hierarchical clustering as explained in methods (Table S6). Functional annotation clustering of each gene set was performed using DAVID. Notably, *SRARP* gene set in breast cancer was highly enrichment for the transcriptional regulatory terms including zinc finger proteins, nuclear hormone receptor, and nuclear receptor corepressor 1 (Tables 5 and S7). Other functional terms in breast cancer included Rab binding domain, calcium‐dependent phospholipid binding, and Heat Shock protein family. Comparatively, *SRARP* gene set in prostate cancer was highly enriched for signaling genes associated with small GTPases, MAPK pathway, protein ubiquitination, and serine phosphorylation/protein kinase activity (Tables 5 and S7). In addition, as observed in breast cancer, prostate gene set was enrichment for zinc finger and chaperone functions. Therefore, *SRARP*‐co‐expressed genes in breast and prostate cancers have similar functional terms associated with transcriptional regulation, small GTPases, and chaperone proteins. However, the degree of enrichment for each function varies between breast and prostate cancers and there is also enrichment for unique pathways in each malignancy.

**Table 5:** Summary of functional annotation clustering for *SRARP*‐co‐expressed gene sets in breast and prostate cancers. Gene sets are identified based on the CC values >0.6 with *SRARP* derived from the average linkage hierarchical clustering in 28 breast cancer and 5 prostate cancer cohorts using the ONCOMINE database. Functional annotation clustering was conducted using DAVID Bioinformatics Resources at a significance level of *P* < 0.05

Terms for *SRARP* gene set in breast cancer	Terms for *SRARP* gene set in prostate cancer
Zinc finger region/transcription factor	Small GTPase signal transduction
Nuclear hormone receptor/GATA‐type	Protein ubiquitination
Nuclear receptor corepressor 1 (NCOR1)	Zinc finger protein
Rab binding domain	MAPK signaling pathway
Calcium‐dependent phospholipid binding	Serine phosphorylation/protein kinase activity
Heat shock protein family	Chaperone

### 3.6 Genomic and epigenetic alterations of *SRARP* and *HSPB7* predict survival

*SRARP* and *HSPB7* genes were next investigated in predicting cancer outcome. In this respect, TCGA Pan‐Cancer datasets were analyzed as explained in methods to examine the association of *SRARP* and *HSPB7* methylation, expression, and mutations with survival across malignancies of multiple tissue origins. In addition, TCGA data from normal solid tissues were separately analyzed. For analysis of each set, TCGA Pan‐Cancer datasets were compiled by combing data from all cohorts, which included DNA methylation beta values, exon expression measured as log2 (RPKM+1)‐transformed exon‐level transcription estimates in RPKM values, gene expression using RNA‐seq results as log2(*x* + 1)‐transformed RSEM values, and somatic mutation data with calls generated using the MuTect method. Survival analysis was performed using Kaplan–Meier curves and the log‐rank test to estimate the survival probability based on DNA methylation, expression, and somatic mutations of *SRARP* and *HSPB7* genes.

**Figure 8:**
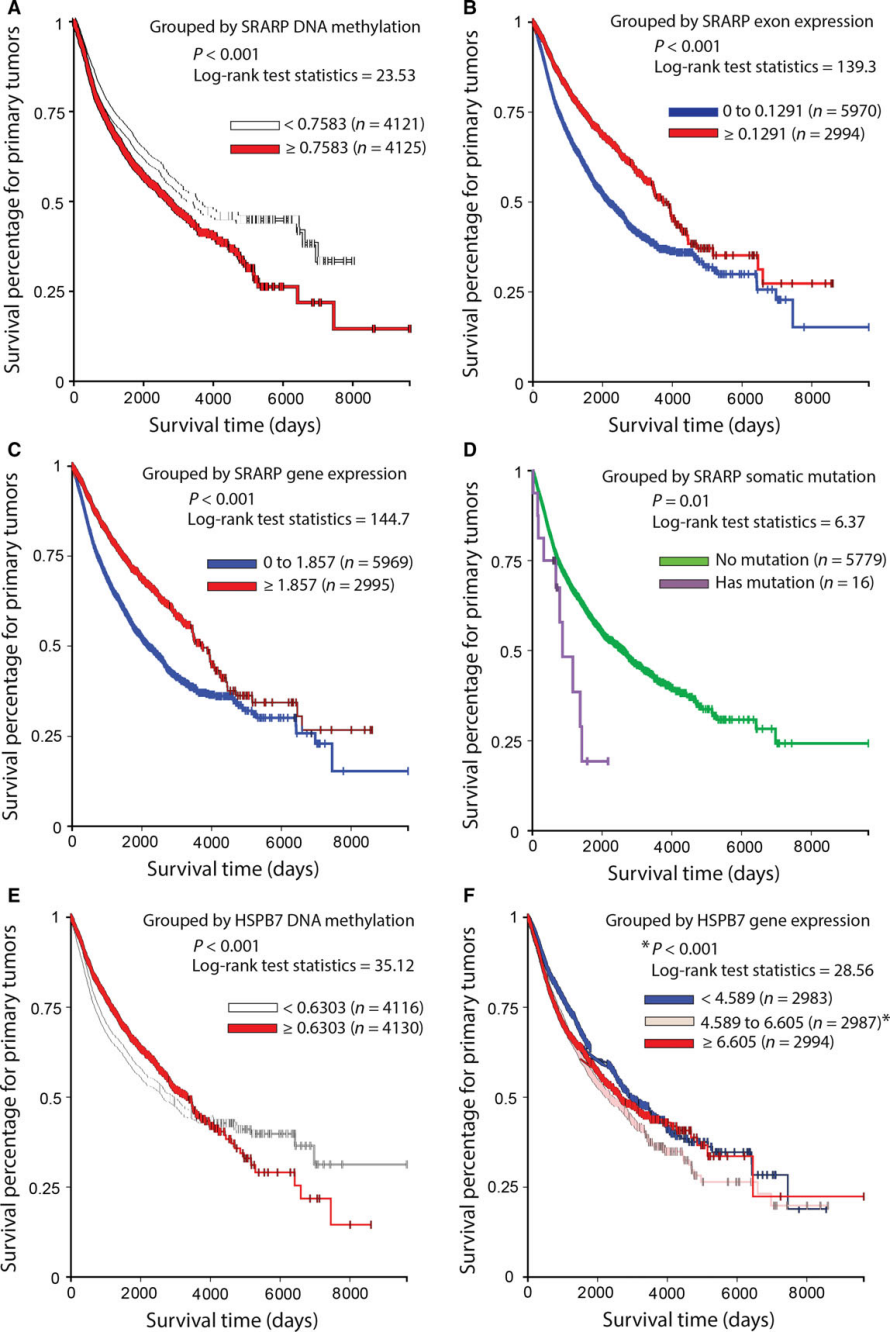
Association of *SRARP* and *HSPB7* methylation, expression, and mutations with survival in primary tumors using TCGA Pan‐Cancer datasets. Survival analysis was performed using Kaplan–Meier curves and the log‐rank test to estimate the survival probability. (A) Survival analysis based on *SRARP* DNA methylation levels in TCGA primary tumors. (B) Survival analysis based on *SRARP* exon expression in TCGA primary tumors. (C) Survival analysis based on *SRARP* gene expression in TCGA primary tumors. (D) Survival analysis based on *SRARP* somatic mutations in TCGA primary tumors. (E) Survival analysis based on *HSPB7* DNA methylation levels in TCGA primary tumors. (F) Survival analysis based on *HSPB7* gene expression in TCGA primary tumors. *P < 0.001 for 4.589 to 6.605 vs. < 4.589 or ≥ 6.605.

The Cancer Genome Atlas Pan‐Cancer DNA methylation and expression datasets in primary tumors were constituted of 8246 and 8964 cases, respectively and had up to 10 000 days (27 years) of follow up. Importantly, *SRARP* DNA methylation strongly predicted survival and a higher *SRARP* methylation level (≥0.7583) was associated with significantly worse survival in primary tumors compared to a lower *SRARP* methylation of <0.7583 (*P* < 0.001, log‐rank test: 23.53; Fig. 8A). In addition, a higher exon expression of *SRARP* (≥0.1291) significantly predicted better survival in primary tumors compared to a lower *SRARP* exon expression of 0 to 0.1291 (*P* < 0.001, log‐rank test: 139.3; Fig. 8B). Gene expression analysis was consistent with these findings, showing that a higher *SRARP* gene expression (≥1.857) significantly predicted improved survival in primary tumors compared to a lower *SRARP* gene expression of 0 to 1.857 (*P* < 0.001, log‐rank test: 144.7; Fig. 8C). Furthermore, the predictive value of *SRARP* somatic mutations for survival was evaluated using TCGA Pan‐Cancer data. Despite the fact that *SRARP* mutations were rare, occurring in 16 of 5795 cases (0.3%), these somatic mutations were significantly associated with poor outcome in primary tumors (*P* = 0.01, log‐rank test: 6.37; Fig. 8D).

Moreover, a higher level of *HSPB7* DNA methylation (≥0.6303) was significantly associated with worse survival in TCGA primary tumors compared to a lower *HSPB7* methylation of <0.6303 (*P* < 0.001, log‐rank test: 35.12; Fig. 8E). However, *HSPB7* expression was not a robust predictor of cancer outcome in TCGA datasets and showed a mixed pattern. In this respect, an intermediate level of *HSPB7* gene expression (4.589 to 6.605) was associated with worse survival compared to gene expression levels of <4.589 or ≥6.605 (*P* < 0.001, log‐rank test: 28.56; Fig. 8F). In addition, *HSPB7* somatic mutations, occurring in 21 of 5795 cases (0.4%), were not a predictor of outcome in primary tumors (Fig. S3).

**Figure 9:**
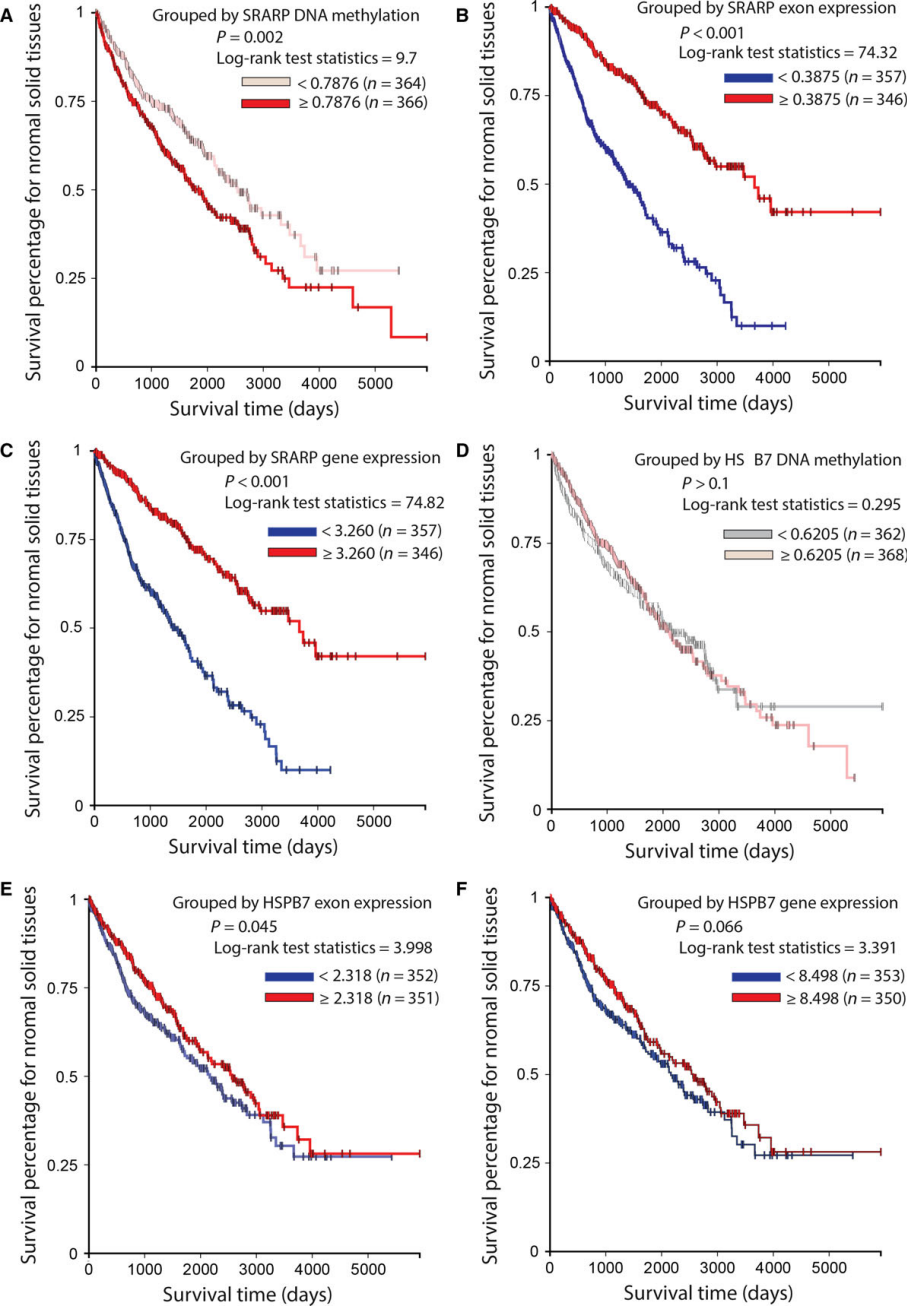
Association of *SRARP* and *HSPB7* methylation and expression with survival in normal solid tissues using TCGA Pan‐Cancer datasets. Survival analysis was performed using Kaplan–Meier curves and the log‐rank test to estimate the survival probability. (A) Survival analysis based on *SRARP* DNA methylation levels in TCGA normal solid tissues. (B) Survival analysis based on *SRARP* exon expression in TCGA normal solid tissues. (C) Survival analysis based on *SRARP* gene expression in TCGA normal solid tissues. (D) Survival analysis based on *HSPB7* DNA methylation levels in TCGA normal solid tissues. (E) Survival analysis based on *HSPB7* exon expression in TCGA normal solid tissues. (F) Survival analysis based on *HSPB7* gene expression in TCGA normal solid tissues.

Next, the association of *SRARP* and *HSPB7* methylation and expression with survival was examined in normal solid tissues derived from TCGA Pan‐Cancer datasets, which mostly constituted of histologically normal tissues adjacent to tumors. Consistent with the results in primary tumors, *SRARP* DNA methylation and expression levels strongly predicted survival in normal solid tissues (Fig. 9A–C). DNA methylation analysis in a total of 730 normal tissues revealed that a higher *SRARP* methylation level (≥0.7876) is associated with significantly worse survival compared to a lower *SRARP* methylation of <0.7876 (*P* = 0.002, log‐rank test: 9.7; Fig. 9A). In addition, a higher exon expression of *SRARP* (≥0.3875) significantly predicted better survival compared to that of <0.3875 in 703 normal tissues (*P* < 0.001, log‐rank test: 74.32; Fig. 9B). A similar result was observed with *SRARP* gene expression data, showing a significantly better survival associated with a higher *SRARP* expression (≥3.260) in normal tissues compared to that of <3.260 (*P* < 0.001, log‐rank test: 74.82; Fig. 9C).

As observed in TCGA primary tumors, *HSPB7* did not consistently predict survival in normal tissues compared to *SRARP*. In this respect, *HSPB7* DNA methylation levels was not significantly associated with survival in normal solid tissues (Fig. 9D). However, a higher level of *HSPB7* exon expression (≥2.318) significantly predicted better survival in 703 normal solid tissues compared to that of <2.318 (*P* = 0.045, log‐rank test: 3.998; Fig. 9E). In contrast, a higher level of *HSPB7* gene expression did not reach statistical significance to predict better outcome (Fig. 9F).

**Figure 10:**
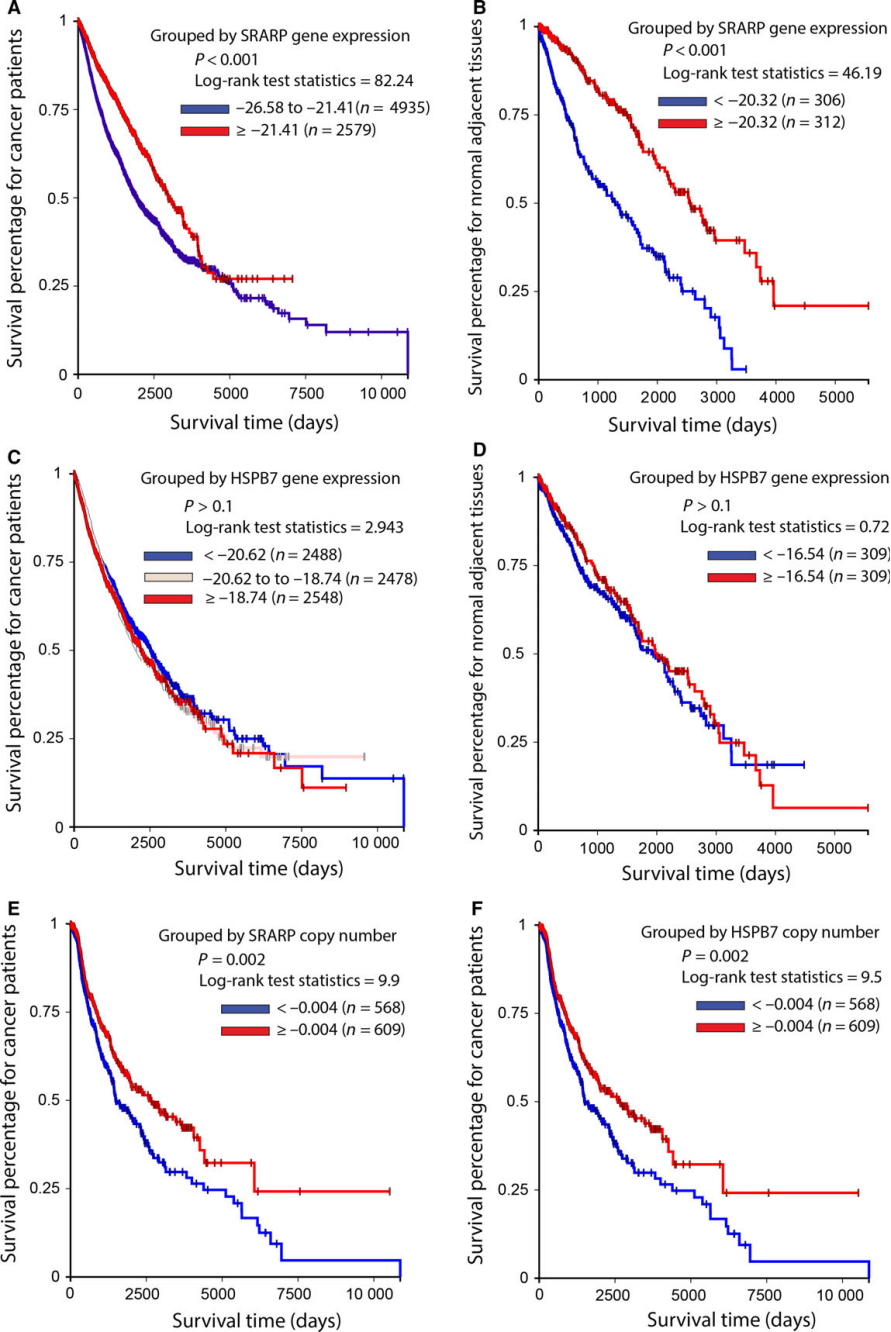
Association of *SRARP* and *HSPB7* gene expression and copy numbers with survival in cancer patients and normal adjacent tissues using ICGC datasets. Survival analysis was performed using Kaplan–Meier curves and the log‐rank test to estimate the survival probability. (A) Survival analysis based on *SRARP* gene expression in ICGC cancer patients. (B) Survival analysis based on *SRARP* gene expression in ICGC normal adjacent tissues. (C) Survival analysis based on *HSPB7* gene expression in ICGC cancer patients. (D) Survival analysis based on *HSPB7* gene expression in ICGC normal adjacent tissues. (E) Survival analysis based on *SRARP* copy number in ICGC cancer patients. (F) Survival analysis based on *HSPB7* copy number in ICGC cancer patients.

Moreover, ICGC datasets were analyzed to further assess the association of *SRARP* and *HSPB7* gene expression with survival in patients with cancer using donor centric data with more than 27 years of follow up. ICGC data from normal adjacent tissues were separately examined. Gene expression results were obtained using RNA‐seq in which expression units are log2 (ICGC‐normalized read count + 1e‐8) values. In addition, survival analysis was carried out in ICGC cohorts using *SRARP* and *HSPB7* copy numbers calculated as log2 (tumor/normal) values. Importantly, *SRARP* gene expression strongly predicted clinical outcome in ICGC cancer patients and normal adjacent tissues using 7514 and 618 cases, respectively (Fig. 10A,B). Of note, a higher *SRARP* gene expression (≥−21.41) was significantly associated with better survival in patients with cancer compared to a lower *SRARP* expression of −26.58 to −21.41 (*P* < 0.001, log‐rank test: 82.24; Fig. 10A). Similarly, a higher gene expression of *SRARP* (≥−20.32) in normal adjacent tissues significantly predicted a better outcome compared to that of <−20.32 (*P* < 0.001, log‐rank test: 46.19; Fig. 10B). However, *HSPB7* gene expression did not significantly predict survival in ICGC cancer patients and normal tissues (*P* > 0.1; Fig. 10C,D).

Finally, the association of *SRARP* and *HSPB7* copy numbers with survival was examined using ICGC datasets in a total of 1177 patients with cancer (Fig. 10E,F). Survival analysis revealed that higher copy numbers of *SRARP* or *HSPB7* (≥−0.004) are significantly associated with improved survival compared to lower copy numbers of <−0.004 (*P* = 0.002, log‐rank test: 9.9 and 9.5 for *SRARP* and *HSPB7*, respectively). As expected, *SRARP* and *HSPB7* copy numbers showed an identical predictive pattern for survival in patients with cancer (Fig. 10E,F).

Collectively, these findings strongly suggest that *SRARP* is a robust predictor of survival in malignancies and normal adjacent tissues. In this respect, higher DNA methylation levels, lower expression, occurrence of somatic mutations, and reduced copy numbers of *SRARP* are significantly associated with worse survival in malignancies. Importantly, higher DNA methylation levels and lower expression of *SRARP* predict reduced survival in normal adjacent tissues. Furthermore, *HSPB7* predicts survival in some datasets and higher DNA methylation levels and lower copy numbers of this gene are associated with worse outcome in malignancies.

## 4 Discussion

Deletions of the distal short arm of chromosome 1 (1p) were first reported in neuroblastomas in 1977 and are present in a broad range of human cancers (Brodeur *et al*., 1977; Henrich *et al*., 2012). It is also established that 1p36 is frequently deleted in malignancies and in particular, 1p36.1 losses occur in 34% of tumors (Henrich *et al*., 2012; Knuutila *et al*., 1999). However, despite extensive studies, there has been limited success for identifying candidate tumor suppressors on chromosome 1p36 (Bagchi and Mills, 2008; Henrich *et al*., 2012). While some of the proposed genes on 1p36 have tumor protective capabilities in specific cellular contexts, none could account for the wide range of tumor types that have been associated with decades of literature documenting 1p36 deletions, suggesting that more than one 1p36 tumor suppressor may exist (Bagchi and Mills, 2008).

The results of the current study strongly suggest that *SRARP* and *HSPB7* are tumor suppressor genes located 5.2 kb apart on 1p36.13. Tumor suppressor functions of SRARP and HSPB7 are supported by the fact that the overexpression of these genes markedly suppresses colony formation and cell viability in cancer cell lines. Notably, this is associated with the downregulation of Akt and ERK signaling and *SRARP* expression inversely correlates with genes that promote cancer cell growth and signal transduction. In addition, the broad pattern of gene‐level deletions and epigenetic inactivation of *SRARP* and *HSPB7* across malignancies of multiple tissue origins is consistent with being tumor suppressor genes in the process of carcinogenesis. Furthermore, genome‐ and epigenome‐wide associations of *SRARP* and *HSPB7* with survival also strongly support their function as tumor suppressors (Figs 8, 9, 10). In particular, this is evident by the fact that DNA hypermethylation, lower gene expression, somatic mutations, and lower copy numbers of *SRARP* are all associated with worse cancer outcome. In addition, DNA hypermethylation and a lower expression of *SRARP* in normal adjacent tissues predict reduced survival, indicating that *SRARP* inactivation is an early event in cancer development. Of note, it is known that the *de novo* methylation of CpG islands and inactivation of tumor suppressors occur early in the process of carcinogenesis and can even be detected in the apparently normal epithelium (Jones and Baylin, 2002; Kazanets *et al*., 2016).

Moreover, *SRARP* and *HSPB7* are gene pairs with highly correlated copy numbers in malignancies. Functional correlations between gene pairs have been previously reported (Arnone *et al*., 2012; Krom and Ramakrishna, 2008). However, as far as author is aware, this is the first time that both genes of any nonhomologous gene pair are shown to be tumor suppressors. In addition, *SPEN*, which is located on 1p36.21‐p36.13, is closely correlated with *SRARP* at the copy number level (Table 1). Interestingly, similar to SRARP, SPEN is also a transcriptional corepressor of nuclear hormone receptors that has a tumor suppressor function in breast cancer (Legare *et al*., 2015, 2017). In view of these facts, the neighboring loci of *SRARP* on chromosome 1p36.13 may be a hotspot region for tumor suppressor genes.

This study demonstrated that there is a strong selection pressure in tumorigenesis to inactivate *SRARP* and *HSPB7*. In this respect, these genes are widely deleted in cancer and are highly regulated by epigenetic mechanisms involving DNA methylation and histone deacetylation. Notably, the majority of normal samples analyzed in TCGA datasets are derived from histologically normal tissues adjacent to tumors that may already have epigenetic changes in tumor suppressor genes. Therefore, comparing *SRARP* and *HSPB7* methylation between tumors and normal adjacent tissues may underestimate their actual hypermethylation levels in cancer. In addition, the fact that *HSPB7* and *SRARP* genes were hypermethylated in fourteen and twelve cancer cell lines, respectively further supports the importance of DNA methylation in the epigenetic regulation of these genes. In addition, *SRARP* expression closely correlates with its methylation level in most tumors and can be predicted using the regression models of its methylation and deacetylation levels in cell lines, suggesting that both of these processes are involved in the epigenetic regulation of *SRARP* in malignancies. However, *HSPB7* expression is better predicted using its deacetylation levels in cell lines compared to methylation, indicating that histone deacetylation may be a key regulatory step for the inactivation of *HSPB7* in cancer.

Of note, there is an increased expression of *SRARP* in breast and prostate tumors compared to their normal tissues that corresponds with its promoter hypomethylation in these cancers. The possibility of epigenetic regulation as an underlying mechanism for this increased expression is further supported by the fact that AR+ cells T‐47D and MFM‐223, which have high levels of *SRARP*, do not demonstrate epigenetic inactivation of this gene. In contrast, AR‐ lines MDA‐MB‐231 and MDA‐MB‐468 have low *SRARP* expression accompanied by marked epigenetic silencing of this gene. Furthermore, a minimum level of AR activity is required for baseline *SRARP* expression in T‐47D and MFM‐223 cells. These findings suggest that the broader effects of AR on the epigenetic regulation of its target genes is the likely underlying mechanism for an increased *SRARP* expression in a subset of breast and prostate tumors. In fact, emerging data suggest that AR activity and androgen‐mediated promoter demethylation contribute to the dynamic regulation of DNA methylation patterns at target genes in prostate tissue and infer further complexity involved in nuclear receptor mediation of transcriptional regulation (Dhiman *et al*., 2015; Hatano *et al*., 2012). Comparatively, *HSPB7* expression is not affected by this mechanism because it is not an AR target gene and due to the fact that *SRARP* and *HSPB7* are convergent gene pairs that do not share their promoter regions.

Furthermore, dual regulatory effects of AR on *SRARP* expression are consistent with the fact that these genes are highly co‐expressed in breast cancer. Although a minimum level of AR activity is required for baseline *SRARP* expression in AR+ cancer cells, higher levels of AR activity lead to another layer of *SRARP* regulation through AR‐mediated suppression of this gene. SRARP, in turn, functions as an AR corepressor to inhibit the reporter activity of androgen response elements and AR‐mediated induction of PIP (Naderi, 2017). It is notable that PIP is a key target of AR that is required for cell cycle progression and acts an effector of AR function in breast cancer (Baniwal *et al*., 2012; Naderi, 2015b; Naderi and Meyer, 2012; Naderi and Vanneste, 2014). In addition, *SRARP* is also repressed by ER activation, providing another layer of negative transcriptional regulation by steroid receptors in breast cancer cells (Luo *et al*., 2016). Collectively, the current study suggests that *SRARP* is inactivated to different degrees in malignancies. Importantly, in breast and prostate cancer cell lines that have low *SRARP* levels, SRARP functions as a tumor suppressor and the overexpression of this gene markedly inhibits colony formation and cell viability.

HSPB7 belongs to the human small heat shock protein (HSPB) family of chaperone proteins that contains a total of eleven family members (Vos *et al*., 2009). *HSPB7* is widely expressed throughout the body with the highest expression observed in the cardiac tissue (Vos *et al*., 2010; Wu *et al*., 2017). Notably, this gene has cardiac protective functions and its mutations result in cardiomyopathies (Wu *et al*., 2017). The fact that in this study a relatively higher *HSPB7* expression was observed in sarcoma may be explained by a common mesodermal origin for all musculoskeletal lineages (Chan *et al*., 2016). In addition, HSPB family, including HSPB7, act protectively on aggregation of several proteins containing an extended polyglutamine (polyQ) stretch, which are linked to a variety of neurodegenerative diseases (Vos *et al*., 2010). In this respect, HSPB7 is the most potent polyQ aggregation suppressor within the HSPB family of chaperones (Vos *et al*., 2010). Furthermore, the only available publication on *HSPB7* in cancer concluded that this gene has a tumor suppressor function in renal cell carcinoma and is epigenetically silenced by hypermethylation in this disease (Lin *et al*., 2014). These findings are in agreement with the current study, suggesting that *HSPB7* is an epigenetically regulated tumor suppressor in multiple malignancies.

In addition, HSPB7 protein contains a HSP20 domain and strongly interacts with the chaperone protein 14‐3‐3 (Figs 2B,C and 7D). HSP20 (HSPB6) is another member of HSPB family that has been shown to interact with the 14‐3‐3 protein (Chernik *et al*., 2007; Sluchanko *et al*., 2011). As a result of this interaction, HSP20 might compete with multiple protein targets of 14‐3‐3 and by this mechanism indirectly affect many intracellular processes (Chernik *et al*., 2007). It has been suggested that some of HSP20 properties can be explained by the interaction of HSP20 with the universal scaffolding and adaptor protein 14‐3‐3 (Chernik *et al*., 2007). Importantly, it is also known that SRARP interacts with the endogenous 14‐3‐3 protein (Naderi, 2017). Of note, 14‐3‐3 is involved in regulating multiple cellular processes and signal transduction pathways by interacting with proteins involved in these processes (Cau *et al*., 2018; Mhawech, 2005). Therefore, an interaction with 14‐3‐3 creates another common molecular feature between HSPB7 and SRARP proteins, which may present an underlying mechanism for their function as tumor suppressors.

The Akt and ERK pathways are central signal transduction mechanisms that are commonly dysregulated in cancers and are interconnected with multiple points of convergence and cross talk (Saini *et al*., 2013). Importantly, SRARP and HSPB7 overexpression lead to a reduction in the relative phosphorylation and/or expression of Akt and ERK in MDA‐MB‐231 and DU‐145 cells (Fig. 7E). These signaling effects of SRARP and HSPB7 correspond with their potent tumor suppressor functions in these two lines (Fig. 6). In view of the integrated nature of Akt and ERK signaling, the downregulation of both these pathways may explain the potent tumor suppressor effects of SRARP and HSPB7 on these cancer cells. Although SRARP and HSPB7 overexpression significantly suppressed the colony formation of A549 cells (Fig. 7A–C), these effects occurred without a corresponding downregulation of Akt and ERK. Therefore, the signaling pathways that are regulated in SRARP‐ and HSPB7‐mediated tumor suppression may vary based on the tissue origin of tumors.

In fact, the effect of SRARP overexpression in reducing Akt and ERK phosphorylation is consistent with the functional association of *SRARP*‐signature genes and SRARP‐co‐expressed gene sets with protein phosphorylation, protein kinase activity, MAPK signaling, and small GTPases (Tables 4 and 5). In addition, the drastic inhibition of Akt protein expression after SRARP overexpression in DU‐145 cell line may be explained by the fact that *SRARP*‐co‐expressed genes in prostate cancer are associated with the protein ubiquitination pathway that regulates protein degradation (Tables 5 and S7). In comparison, protein ubiquitination is not associated with *SRARP*‐co‐expressed genes in breast cancer. Therefore, *SRARP* expression is associated with transcriptional regulation, small GTPases, and chaperone proteins in both breast and prostate cancers; however, SRARP also correlates with unique pathways in each malignancy. Of note, 14‐3‐3 is known to regulate both the Akt and ERK signaling pathways at multiple levels (Ajjappala *et al*., 2009; Gomez‐Suarez *et al*., 2016; Mhawech, 2005). Collectively, these findings suggest that SRARP and HSPB7 interactions with 14‐3‐3 protein and the regulation of Akt and ERK may be interconnected.

It is important to highlight that the findings of this study may have relevance to 1p36 deletion syndrome. Deletions of chromosome 1p36 affect approximately 1 in 5000 newborns and are the most common terminal deletions in humans (Jordan *et al*., 2015). This syndrome has a broad range of anomalies that include mental retardation, developmental delay, hearing and vision impairments, seizures, growth impairment, and congenital heart defects (Gajecka *et al*., 2007; Jordan *et al*., 2015). Furthermore, 1p36 deletion syndrome has also been associated with the occurrence of neuroblastoma and paraganglioma (Anderson *et al*., 2001; Murakoshi *et al*., 2017). The clinical and genetic heterogeneity seen among individuals with 1p36 deletions present a significant challenge and, in part, this is because the genes that contribute to most 1p36‐related phenotypes have yet to be identified [54]. Notably, chromosome 1p36.13, which contains *SRARP* and *HSPB7* genes, is one of the deleted regions in 1p36 syndrome and has been suggested as a critical region for congenital heart defects in this syndrome (Jordan *et al*., 2015; Zaveri *et al*., 2014). In view of an established protective function for *HSPB7* in the cardiac tissue, this gene may be involved in the cardiovascular phenotype of 1p36 deletion syndrome. Furthermore, due to the proximity of *SRARP* and *HSPB7* genes, they are likely to be deleted in the same subset of patients, which suggests they may have a combined impact on the disease phenotype that warrants investigation.

Finally, the robust association of *SRARP* inactivation with worse survival in malignancies and normal solid tissues has important translational implications. This association indicates that *SRARP* inactivation by deletion, epigenetic silencing, or mutations may occur in a large subset of malignancies and has a detrimental effect on cancer outcome. In addition, *SRARP* predictive value in normal solid tissues indicates that the inactivation of this tumor suppressor is an early event in carcinogenesis occurring in apparently normal epithelium. Therefore, DNA methylation and expression levels of *SRARP* in addition to its copy number and somatic mutations, either alone or in combination, may be valuable predictors of survival in malignancies. Importantly, *SRARP* methylation and expression levels in normal solid tissues may also have diagnostic applications in the workup of biopsy samples with histologically normal tissues that contain *SRARP* inactivation. In these cases, molecular evidence for the presence of *SRARP* inactivation may justify further investigations or a closer follow up to detect early malignancies.

## 5 Conclusions

This study suggests that *SRARP* and *HSPB7* are gene pairs on 1p36.13 that have tumor suppressor functions and are highly regulated by gene‐level deletions and epigenetic silencing across malignancies of multiple tissue origins. Of note, tumor suppressor functions of SRARP and HSPB7 are associated with the downregulation of Akt and ERK signaling in cancer cells. In addition, SRARP and HSPB7 both interact with the 14‐3‐3 protein, presenting a possible underlying mechanism for their molecular functions. Importantly, *SRARP* inactivation is an early event in carcinogenesis that is strongly associated with worse survival in both malignancies and normal adjacent tissues and has potential translational applications.

## Author contributions

AN conceived the study, performed the bioinformatics analysis, carried out the experiments, interpreted the data, and drafted the manuscript.

## Supporting information


**Fig. S1.** Box plots to show *HSPB7* and *SRARP* expression following CoCl2 treatment and heat shock in T‐47D and MFM‐223 cell lines.

**Fig. S2.** Graphs for regression models to predict *SRARP* and *HSPB7* expression based on their epigenetic regulation.

**Fig. S3.** Kaplan–Meier curve to estimate the association of *HSPB7* somatic mutations with survival in primary tumors.

**Table S1.** A table presenting copy number correlation values between *SRARP* and *HSPB7* genes in malignancies.

**Table S2.** List of genes that have highly correlated copy numbers with *SRARP* at a correlation coefficient cutoff of > 0.95 across 37 datasets in malignancies.

**Table S3.** A table showing the promoter methylation values for *SRARP* and *HSPB7* genes in tumors and matched normal samples.

**Table S4.** List of *SRARP*‐signature genes based on Pearson correlation coefficient (PCC) values of >0.6 or <−0.6 (*P* < 0.001) with *SRARP* expression in breast cancer.

**Table S5.** Functional annotation clustering of *SRARP*‐signature genes based on positive (a) or inverse (b) correlations with *SRARP* expression in 50 breast cancer cell lines.

**Table S6.** List of *SRARP*‐co‐expressed genes in breast and prostate cancers based on the correlation values of >0.6 (*P* ≤ 0.0001) derived from the average linkage hierarchical clustering.

**Table S7.** Functional annotation clustering of *SRARP*‐co‐expressed genes across 28 breast cancer (a) and 5 prostate cancer (b) datasets.
