# Supplementary material for: SRARP and HSPB7 are epigenetically regulated gene pairs that function as tumor suppressors and predict clinical outcome in malignancies
Source: Mol Oncol. 2018 Apr 16;12(5):724–55. doi: 10.1002/1878-0261.12195 (PMC5928383; doi:10.1002/1878-0261.12195)
Supplement: Supplementary file 1 — Fig. S1. Box plots to show HSPB7 and SRARP expression following CoCl2 treatment and heat shock in T‐47D and MFM‐223 cell lines. [file MOL2-12-724-s001.docx]

**Figure S1**


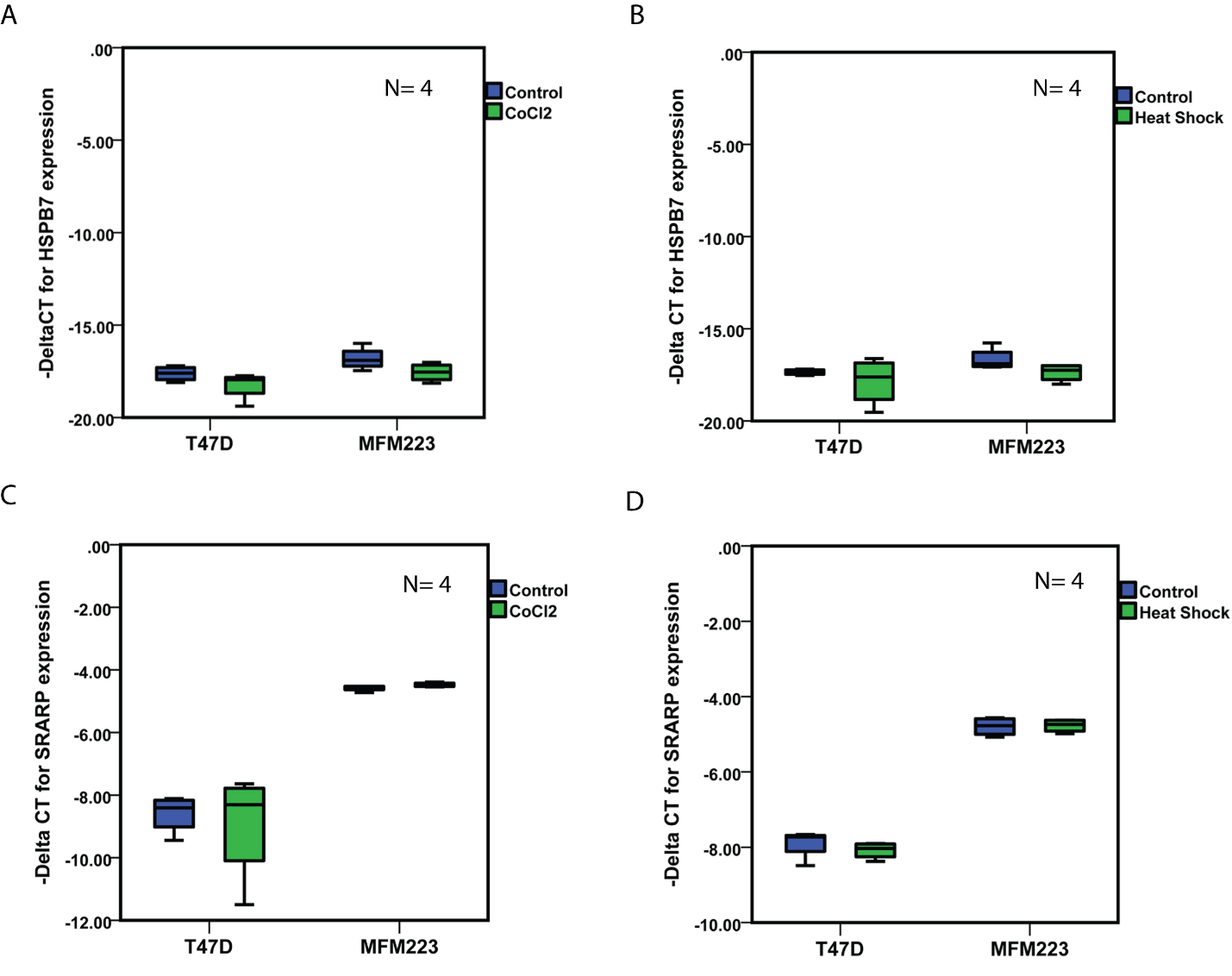


**Figure S1.** Box plots to show HSPB7 and SRARP expression following CoCl2 treatment and heat shock in T-47D and MFM-223 breast cancer cell lines. (A) –Delta CT (CT:cycle threshold) values for HSPB7 expression using qRT-PCR following CoCl2 treatment at 100 µM concetration for 24h. (B) –Delta CT values for HSPB7 expression using qRT-PCR following heat shock at 42 °C. (C) –Delta CT values for SRARP expression using qRT-PCR following CoCl2 treatment at 100 µM concetration for 24h. (D) –Delta CT values for SRARP expression using qRT-PCR following heat shock at 42 °C.
