# Supplementary material for: SRARP and HSPB7 are epigenetically regulated gene pairs that function as tumor suppressors and predict clinical outcome in malignancies
Source: Mol Oncol. 2018 Apr 16;12(5):724–55. doi: 10.1002/1878-0261.12195 (PMC5928383; doi:10.1002/1878-0261.12195)
Supplement: Supplementary file 2 — Fig. S2. Graphs for regression models to predict SRARP and HSPB7 expression based on their epigenetic regulation. [file MOL2-12-724-s002.docx]

**Figure S2**

**
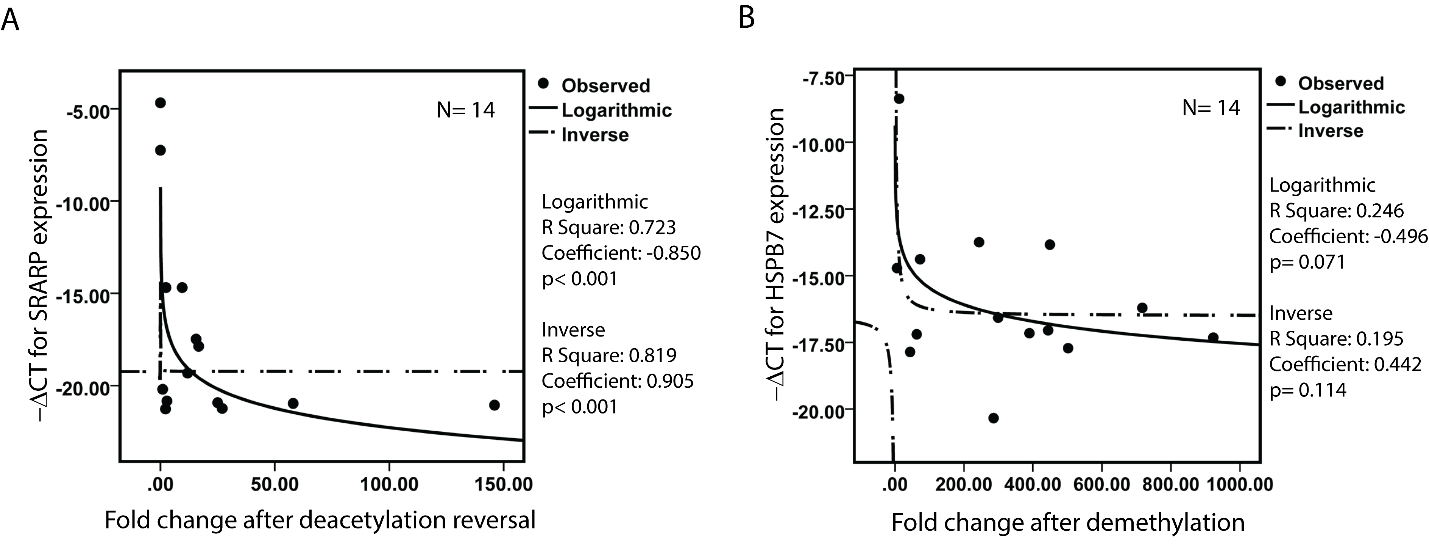
**

**Figure S2.** Regression models to predict SRARP and HSPB7 expression based on their epigenetic regulation. SRARP and HSPB7 mRNA expression with and without epigenetic modulations were measured using qRT-PCR in fourteen cancer cell lines. (A) Logarithmic and inverse regression models to predict SRARP expression after histone deacetylation reversal using Trichostatin A (TSA). (B) Logarithmic and inverse regression models to predict HSPB7 expression after DNA demethylation using 5-aza-2'-deoxycytidine (AZA). -ΔCT is -Δ Cycle Threshold value for gene expression. R Square, standardized coefficients, and p values for each model are shown.
