# Supplementary material for: SRARP and HSPB7 are epigenetically regulated gene pairs that function as tumor suppressors and predict clinical outcome in malignancies
Source: Mol Oncol. 2018 Apr 16;12(5):724–55. doi: 10.1002/1878-0261.12195 (PMC5928383; doi:10.1002/1878-0261.12195)
Supplement: Supplementary file 3 — Fig. S3. Kaplan–Meier curve to estimate the association of HSPB7 somatic mutations with survival in primary tumors. [file MOL2-12-724-s003.docx]

**Figure S3**


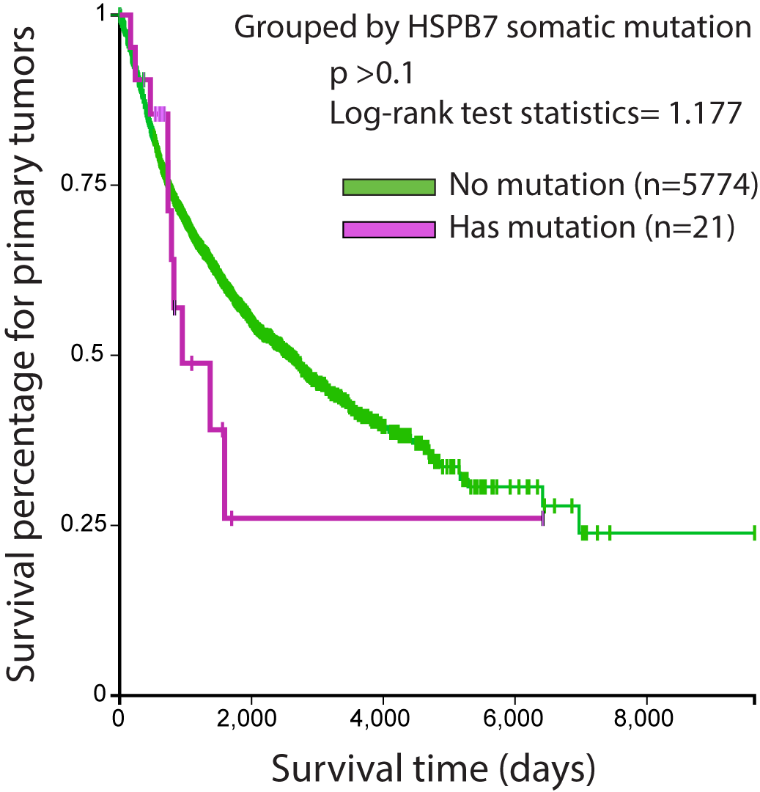


**Figure S3.** Kaplan-Meier curve to estimate the association of HSPB7 somatic mutations with survival in primary tumors. TCGA pan-cancer datasets were accessed using the UCSC Xena browser and bioinformatics tool (<https://xenabrowser.net/>). Significance is calculated using the Log-rank test.
