# Supplementary material for: SRARP and HSPB7 are epigenetically regulated gene pairs that function as tumor suppressors and predict clinical outcome in malignancies
Source: Mol Oncol. 2018 Apr 16;12(5):724–55. doi: 10.1002/1878-0261.12195 (PMC5928383; doi:10.1002/1878-0261.12195)
Supplement: Supplementary file 4 — Table S1. A table presenting copy number correlation values between SRARP and HSPB7 genes in malignancies. [file MOL2-12-724-s004.docx]

**Table S1.** Copy number correlations between SRARP and HSPB7 genes across malignancies analyzed using *ONCOMINE database*. Study name and reference, correlation coefficient (p≤ 0.0001), tissue type, and sample size are shown for each study. Collectively, these results include a total of 12,767 samples across 37 cancer datasets.

| **Study** | **Correlation** | **Tissue** | **Sample size** |
| --- | --- | --- | --- |
| Beroukhim [1] | 1 | Brain | 187 |
| Kotliarov [2] | 1 | Brain | 178 |
| George [3] | 1 | Brain | 25 |
| TCGA [4] | 0.999 | Brain | 1531 |
| Chen [5] | 1 | Brain | 239 |
| TCGA [4] | 0.99 | Breast | 1602 |
| Nikolsky [6] | 0.953 | Breast | 191 |
| Curtis [7] | 0.988 | Breast | 1992 |
| Reid [8] | 1 | Colon | 48 |
| Firestein [9] | 1 | Colorectal | 123 |
| Kurashina [10] | 1 | Colon | 188 |
| TCGA 2 [4] | 0.967 | Colorectal | 881 |
| Salvesen [11] | 1 | Endometrium | 84 |
| TCGA [4] | 1 | Endometrium | 726 |
| Yoshida [12] | 1 | Leukemia | 58 |
| Maser [13] | 1 | Leukemia | 18 |
| Kuhn [14] | 1 | Leukemia | 516 |
| TCGA 2 [4] | 0.954 | Leukemia | 392 |
| Chiang 2 [15] | 1 | Liver | 197 |
| Guichard 2 [16] | 1 | Liver | 52 |
| Guichard [16] | 1 | Liver | 185 |
| TCGA [4] | 1 | Liver | 212 |
| Bass [17] | 1 | Lung | 47 |
| Ramos [18] | 1 | Lung | 30 |
| Rinaldi [19] | 1 | Lymphoma | 218 |
| Braggio [20] | 1 | Lymphoma | 114 |
| Brachmann [21] | 1 | Mesothelioma | 26 |
| Jaiswal [22] | 1 | Multi-cancer | 81 |
| Neale 2 [23] | 1 | Multi-cancer | 59 |
| Beroukhim [21] | 0.999 | Multi-cancer | 698 |
| Etemadmoghadam [24] | 1 | Ovarian | 118 |
| Harada [25] | 1 | Pancreas | 28 |
| TCGA [4] | 1 | Pancreas | 100 |
| Taylor [26] | 0.982 | Prostate | 231 |
| TCGA 2 [4] | 0.979 | Renal | 1071 |
| Beroukhim [27] | 1 | Renal | 111 |
| Barretina 2 [28] | 1 | Sarcoma | 210 |
|  |  |  |  |

**References for Table S1**

[1] Beroukhim R, Getz G, Nghiemphu L, Barretina J, Hsueh T, Linhart D, Vivanco I, Lee JC, Huang JH, Alexander S, et al. (2007). Assessing the significance of chromosomal aberrations in cancer: methodology and application to glioma *Proc Natl Acad Sci U S A* **104**, 20007-20012.

[2] Kotliarov Y, Steed ME, Christopher N, Walling J, Su Q, Center A, Heiss J, Rosenblum M, Mikkelsen T, Zenklusen JC, et al. (2006). High-resolution global genomic survey of 178 gliomas reveals novel regions of copy number alteration and allelic imbalances *Cancer Res* **66**, 9428-9436.

[3] George RE, Attiyeh EF, Li S, Moreau LA, Neuberg D, Li C, Fox EA, Meyerson M, Diller L, Fortina P, et al. (2007). Genome-wide analysis of neuroblastomas using high-density single nucleotide polymorphism arrays *PLoS One* **2**, e255.

[4] Grossman RL, Heath AP, Ferretti V, Varmus HE, Lowy DR, Kibbe WA, Staudt LM (2016). Toward a Shared Vision for Cancer Genomic Data *N Engl J Med* **375**, 1109-1112.

[5] Chen Y, Takita J, Choi YL, Kato M, Ohira M, Sanada M, Wang L, Soda M, Kikuchi A, Igarashi T, et al. (2008). Oncogenic mutations of ALK kinase in neuroblastoma *Nature* **455**, 971-974.

[6] Nikolsky Y, Sviridov E, Yao J, Dosymbekov D, Ustyansky V, Kaznacheev V, Dezso Z, Mulvey L, Macconaill LE, Winckler W, et al. (2008). Genome-wide functional synergy between amplified and mutated genes in human breast cancer *Cancer Res* **68**, 9532-9540.

[7] Curtis C, Shah SP, Chin SF, Turashvili G, Rueda OM, Dunning MJ, Speed D, Lynch AG, Samarajiwa S, Yuan Y, et al. (2012). The genomic and transcriptomic architecture of 2,000 breast tumours reveals novel subgroups *Nature* **486**, 346-352.

[8] Reid JF, Gariboldi M, Sokolova V, Capobianco P, Lampis A, Perrone F, Signoroni S, Costa A, Leo E, Pilotti S, et al. (2009). Integrative approach for prioritizing cancer genes in sporadic colon cancer *Genes Chromosomes Cancer* **48**, 953-962.

[9] Firestein R, Bass AJ, Kim SY, Dunn IF, Silver SJ, Guney I, Freed E, Ligon AH, Vena N, Ogino S, et al. (2008). CDK8 is a colorectal cancer oncogene that regulates beta-catenin activity *Nature* **455**, 547-551.

[10] Kurashina K, Yamashita Y, Ueno T, Koinuma K, Ohashi J, Horie H, Miyakura Y, Hamada T, Haruta H, Hatanaka H, et al. (2008). Chromosome copy number analysis in screening for prognosis-related genomic regions in colorectal carcinoma *Cancer Sci* **99**, 1835-1840.

[11] Salvesen HB, Carter SL, Mannelqvist M, Dutt A, Getz G, Stefansson IM, Raeder MB, Sos ML, Engelsen IB, Trovik J, et al. (2009). Integrated genomic profiling of endometrial carcinoma associates aggressive tumors with indicators of PI3 kinase activation *Proc Natl Acad Sci U S A* **106**, 4834-4839.

[12] Yoshida K, Sanada M, Shiraishi Y, Nowak D, Nagata Y, Yamamoto R, Sato Y, Sato-Otsubo A, Kon A, Nagasaki M, et al. (2011). Frequent pathway mutations of splicing machinery in myelodysplasia *Nature* **478**, 64-69.

[13] Maser RS, Choudhury B, Campbell PJ, Feng B, Wong KK, Protopopov A, O'Neil J, Gutierrez A, Ivanova E, Perna I, et al. (2007). Chromosomally unstable mouse tumours have genomic alterations similar to diverse human cancers *Nature* **447**, 966-971.

[14] Kuhn MW, Radtke I, Bullinger L, Goorha S, Cheng J, Edelmann J, Gohlke J, Su X, Paschka P, Pounds S, et al. (2012). High-resolution genomic profiling of adult and pediatric core-binding factor acute myeloid leukemia reveals new recurrent genomic alterations *Blood* **119**, e67-75.

[15] Chiang DY, Villanueva A, Hoshida Y, Peix J, Newell P, Minguez B, LeBlanc AC, Donovan DJ, Thung SN, Sole M, et al. (2008). Focal gains of VEGFA and molecular classification of hepatocellular carcinoma *Cancer Res* **68**, 6779-6788.

[16] Guichard C, Amaddeo G, Imbeaud S, Ladeiro Y, Pelletier L, Maad IB, Calderaro J, Bioulac-Sage P, Letexier M, Degos F, et al. (2012). Integrated analysis of somatic mutations and focal copy-number changes identifies key genes and pathways in hepatocellular carcinoma *Nat Genet* **44**, 694-698.

[17] Bass AJ, Watanabe H, Mermel CH, Yu S, Perner S, Verhaak RG, Kim SY, Wardwell L, Tamayo P, Gat-Viks I, et al. (2009). SOX2 is an amplified lineage-survival oncogene in lung and esophageal squamous cell carcinomas *Nat Genet* **41**, 1238-1242.

[18] Ramos AH, Dutt A, Mermel C, Perner S, Cho J, Lafargue CJ, Johnson LA, Stiedl AC, Tanaka KE, Bass AJ, et al. (2009). Amplification of chromosomal segment 4q12 in non-small cell lung cancer *Cancer Biol Ther* **8**, 2042-2050.

[19] Rinaldi A, Mian M, Chigrinova E, Arcaini L, Bhagat G, Novak U, Rancoita PM, De Campos CP, Forconi F, Gascoyne RD, et al. (2011). Genome-wide DNA profiling of marginal zone lymphomas identifies subtype-specific lesions with an impact on the clinical outcome *Blood* **117**, 1595-1604.

[20] Braggio E, Dogan A, Keats JJ, Chng WJ, Huang G, Matthews JM, Maurer MJ, Law ME, Bosler DS, Barrett M, et al. (2012). Genomic analysis of marginal zone and lymphoplasmacytic lymphomas identified common and disease-specific abnormalities *Mod Pathol* **25**, 651-660.

[21] Beroukhim R, Mermel CH, Porter D, Wei G, Raychaudhuri S, Donovan J, Barretina J, Boehm JS, Dobson J, Urashima M, et al. (2010). The landscape of somatic copy-number alteration across human cancers *Nature* **463**, 899-905.

[22] Jaiswal BS, Janakiraman V, Kljavin NM, Chaudhuri S, Stern HM, Wang W, Kan Z, Dbouk HA, Peters BA, Waring P, et al. (2009). Somatic mutations in p85alpha promote tumorigenesis through class IA PI3K activation *Cancer Cell* **16**, 463-474.

[23] Neale G, Su X, Morton CL, Phelps D, Gorlick R, Lock RB, Reynolds CP, Maris JM, Friedman HS, Dome J, et al. (2008). Molecular characterization of the pediatric preclinical testing panel *Clin Cancer Res* **14**, 4572-4583.

[24] Etemadmoghadam D, deFazio A, Beroukhim R, Mermel C, George J, Getz G, Tothill R, Okamoto A, Raeder MB, Harnett P, et al. (2009). Integrated genome-wide DNA copy number and expression analysis identifies distinct mechanisms of primary chemoresistance in ovarian carcinomas *Clin Cancer Res* **15**, 1417-1427.

[25] Harada T, Chelala C, Bhakta V, Chaplin T, Caulee K, Baril P, Young BD, Lemoine NR (2008). Genome-wide DNA copy number analysis in pancreatic cancer using high-density single nucleotide polymorphism arrays *Oncogene* **27**, 1951-1960.

[26] Taylor BS, Schultz N, Hieronymus H, Gopalan A, Xiao Y, Carver BS, Arora VK, Kaushik P, Cerami E, Reva B, et al. (2010). Integrative genomic profiling of human prostate cancer *Cancer Cell* **18**, 11-22.

[27] Beroukhim R, Brunet JP, Di Napoli A, Mertz KD, Seeley A, Pires MM, Linhart D, Worrell RA, Moch H, Rubin MA, et al. (2009). Patterns of gene expression and copy-number alterations in von-hippel lindau disease-associated and sporadic clear cell carcinoma of the kidney *Cancer Res* **69**, 4674-4681.

[28] Barretina J, Taylor BS, Banerji S, Ramos AH, Lagos-Quintana M, Decarolis PL, Shah K, Socci ND, Weir BA, Ho A, et al. (2010). Subtype-specific genomic alterations define new targets for soft-tissue sarcoma therapy *Nat Genet* **42**, 715-721.
